# Supplementary material for: Nucleophilicity at copper(-I) in a compound with a Cu–Mg bond
Source: Nat Commun. 2025 Jan 28;16:1101. doi: 10.1038/s41467-025-56544-z (PMC11775243; doi:10.1038/s41467-025-56544-z)
Supplement: Supplementary file 1 — Supplementary Information [file 41467_2025_56544_MOESM1_ESM.pdf]

Supplementary Information for

Nucleophilicity at copper(-I) in a compound with a Cu–Mg bond

Ross A. Jackson[1], Nicholas J. Evans[1], Dawid J. Babula[1,2], Thomas M. Horsley Downie[1], Rex S. C. Charman[1], Samuel E. Neale[1], Mary F. Mahon[1], David J. Liptrot[1]*

Supplementary Note 1 2
Supplementary Methods 2
(6-Dipp)CuOEt 2
$[LMg]_2$ 2
LZnI 3
[(6-Dipp)Cu(μ_2-OEt)$_2$MgL] (1) 3
[(6-Dipp)CuMgL] (2) 3
[(6-Dipp)Cu{μ-H}$_2$MgL] (3) 4
[(6-Dipp)CuC(NCy)$_2$MgL] (4) 4
[(6-Dipp)CuSiMe$_2$Ph] (5) 5
[(6-Dipp)CuFeCp(CO)$_2$] (6) 5
[(6-Dipp)CuZnL] (7) 6
NMR Scale Reactions 6
[(6-Dipp)CuMgL] (2) + ^{t}BuOH 6
[(6-Dipp)CuMgL] (2) + HSi(Me)$_2$Ph 7
[(6-Dipp)CuMgL] (2) + PhCl 7
[(6-Dipp)CuMgL] (2) + Ph$_2$POMe 7
[(6-Dipp)CuMgL] (2) + CpFe(CO)$_2$I 7
Supplementary Note 2 9
In-situ monitoring of [(6-Dipp)CuMgL] (2) + LZnI 9
Supplementary Note 3 10
Supplementary Figures 22
NMR Spectra 22
NMR Scale Reactions 33
IR Spectra 38
Supplementary Note 4 41
Computational Details 41
QTAIM Contour Plots & Tabulated Data 41
MO and NBO Data 42
IRC-Charge analysis 43
References 45

Supplementary Note 1

All reactions involving air- and moisture-sensitive compounds were carried out under an argon atmosphere using standard Schlenk line and glovebox techniques. NMR experiments using air-sensitive compounds were conducted in J. Young's tap NMR tubes prepared and sealed in a glovebox under argon. Milling reactions were performed in a Retsch PM100 ball mill, in a 25 mL zirconia jar 1/3 filled with 0.5 mm diameter zirconia balls (80 g), and sealed with a Retsch Safety Clamp. All NMR data were acquired at 298 K on an Agilent ProPulse/Bruker Avance NEO instrument for ^{1}H (500 MHz), ^{2}H (77 MHz) and ^{13}C (126 MHz) or Avance NEO 400 instrument for ^{1}H (400 MHz) and ^{13}C (100 MHz). ^{1}H and ^{13}C NMR spectra were referenced using residual C_6D_6 solvent resonances. Data was processed using MestReNova software. Elemental analyses were performed by Elemental Microanalysis Ltd., Okehampton, Devon, U.K. ICP-OES was performed by Butterworth Laboratories Ltd., Teddington, U.K. Benzene was dried over sodium and stored over 4 Å molecular sieves. Hexane and pentane were purified using an MBraun Solvent Purification System and stored over 4 Å molecular sieves. C_6D_6 was dried over a potassium mirror prior to vacuum transfer into a sealed ampoule and stored in the glove box under argon. Starting materials were purchased from standard suppliers and used without further purification unless otherwise stated. $[LMg]_2$ [1] and LZnI[2] were synthesised according to literature conditions as described below.

NMR Spectroscopy Multiplet Abbreviations

s	singlet
d	doublet
t	triplet
q	quartet
p	pentet
h	heptet
m	multiplet

Supplementary Methods

(6-Dipp)CuOEt

(6-Dipp)CuOEt was prepared via an adapted literature procedure.[3]

(6-Dipp)HBr (3.0000 g, 1 eq, 6.1786 mmol), CuCl (611.7 mg, 1 eq, 6.1786 mmol) and milling balls were placed in a milling jar and suspended in toluene (10 mL). The mixture was sealed inside the glove box and milled for 30 minutes (500 rpm). The mixture was opened inside a glove box and NaOEt (1.261 g, 3 eq, 18.54 mmol) was added. The milling jar was resealed and milled at 500 rpm (3 × 30-minute intervals). The resulting slurry was transferred to a frit and extracted with toluene (60 mL). The resulting clear solution was dried *in vacuo* yielding an off-white powder. Crystals suitable for SCXRD and EA were crystalised from a saturated toluene solution.

^{1}H NMR (500 MHz, C_6D_6): δ 7.19 (apparent t (overlaps with solvent), *J* = 7.7 Hz, 2H, Ar$\underline{H}$), 7.07 (d, *J* = 7.7 Hz, 4H, Ar$\underline{H}$), 4.01 (q, *J* = 6.7 Hz, 2H, CuOC$\underline{H}_2$CH$_3$), 3.00 (h, *J* = 7.0 Hz, 4H, iPr C$\underline{H}$)), 2.69 (t, *J* = 5.9 Hz, 4H, NC$\underline{H}_2$CH$_2$), 1.47 (d, *J* = 6.9 Hz, 12H, iPr C$\underline{H}_3$), 1.44 (m, 2H, NCH$_2$C$\underline{H}_2$, overlaps with 1.47), 1.15 (d, *J* = 6.9 Hz, 12H), 0.91 (t, *J* = 6.7 Hz, 3H).

$^{13}C\{^{1}H\}$ NMR (125 MHz, C_6D_6): δ 203.3 ($\underline{C}$Cu), 145.7(Ar$\underline{C}$), 142.5 (Ar$\underline{C}$), 129.3 (Ar$\underline{C}$), 124.8 (Ar$\underline{C}$), 63.1 (Cu(O$\underline{C}$H$_2$CH$_3$), 46.1 (N$\underline{C}$H$_2$CH$_2$), 28.9 (iPr $\underline{C}$H), 25.1 (iPr $\underline{C}$H$_3$), 24.6 (Cu(OCH$_2\underline{C}$H$_3$), 20.4 (NCH$_2\underline{C}$H$_2$).

Anal. Calc. for $C_{30}H_{45}CuN_2O$: C, 70.21; H, 8.84; N 5.46 %. Found: C, 70.80; H, 8.68; N, 4.98 %.

$[LMg]_2$

In a Schlenk flask LMgI(OEt$_2$) (4.0000 g, 1.0 eq, 6.221 mmol) and Na/NaCl (20 % wt of Na) (858.1 mg, 1.2 eq, 7.465 mmol) were mixed together. Toluene (100 mL) and diethyl ether (10 mL) were then added. The resulting dark black suspension was filtered, yielding a vibrant yellow solution. This was concentrated *in vacuo* and washed with pentane (2 × 15 mL). The resulting yellow powder was identified by ^{1}H NMR spectroscopy as the product $[LMg]_2$ (2.5565 g, 93 %).

^{1}H NMR (500 MHz, C_6D_6): δ 7.10 – 7.04 (m, 12H, Ar$\underline{H}$), 4.82 (s, 2H, NC(CH$_3$)C$\underline{H}$), 3.07 (h, *J* = 6.8 Hz, 8H, iPr C$\underline{H}$), 1.54 (s, 12H, NC(C$\underline{H}_3$)CH), 1.16 (d, *J* = 6.9 Hz, 24H, iPr C$\underline{H}_3$), 0.98 (d, *J* = 6.9 Hz, 24H, iPr C$\underline{H}_3$).

LZnI

In a Schlenk flask LH (2.5466 g, 1.0 eq, 6.0826 mmol) was dissolved in THF (20 mL). A KN(SiMe$_3$)$_2$ (1.2741 g, 1.05 eq, 6.3867 mmol) solution in THF (20 mL) was added generating a clear yellow solution. The solution was allowed to stir for 2 hours at RT. In a separate Schlenk flask, ZnI$_2$ (1.9415 g, 1 eq, 6.0826 mmol) was suspended in THF (20 mL) and cooled to 0 °C. The yellow solution was added dropwise to the suspension of ZnI$_2$, which slowly began to form a white precipitate. After the ZnI$_2$ suspension was added, the solution was allowed to warm gently to RT and stirred overnight. The suspension was concentrated *in vacuo* before extracted with hot toluene (2 × 25 mL). The resulting yale yellow solution was concentrated *in vacuo* and washed with hexane (2 × 15 mL) and dried *in vacuo* to give a white solid (1.9781 g, 53 %).

^{1}H NMR (500 MHz, C$_6$D$_6$): δ 7.14 – 7.08 (m, 6H, Ar$\underline{\text{H}}$), 4.98 (s, 1H, NC(CH$_3$)C$\underline{\text{H}}$), 3.10 (h, *J* = 6.9 Hz, 4H, iPr C$\underline{\text{H}}$), 1.67 (s, 6H, NC(C$\underline{\text{H}}_3$)CH), 1.36 (d, *J* = 6.9 Hz, 12H, iPr C$\underline{\text{H}}_3$), 1.13 (d, *J* = 6.9 Hz, 12H, iPr C$\underline{\text{H}}_3$).

[(6-Dipp)Cu(μ$_2$-OEt)$_2$MgL] (1)

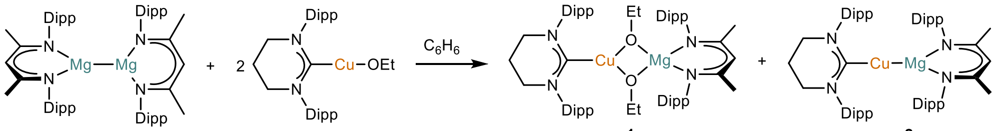

1
Yield: 99%

2
Yield: 34%

In a Schlenk flask to (LMg)$_2$ (660.0 mg, 1.0 eq, 746.7 µmol) and (6-Dipp)CuOEt (790 mg, 2.1 eq, 1.54 mmol) was added benzene (15 mL) and the resultant slurry stirred. After 2 hours additional (6-Dipp)CuOEt (76.7 mg, 0.2 eq, 149.3 µmol) was added and stirring was continued. After two further hours of stirring the volatiles were removed. The resulting crude solid was triturated with pentane (2 × 15 mL). The product was washed twice with pentane and volatiles removed *in vacuo*, yielding a red-grey powder. Retrieved 797.4 mg (99% at 93% purity). Suitable crystals for SCXRD were grown from a C$_6$D$_6$/pentane mixture at −30 °C.

^{1}H NMR (500 MHz, C$_6$D$_6$): δ 7.23 – 7.17 (m, 8H, Ar$\underline{\text{H}}$), 7.03 (d, J = 7.7 Hz, 4H, Ar$\underline{\text{H}}$), 4.92 (s, 1H, NC(CH$_3$)C$\underline{\text{H}}$), 3.62 (h, J = 6.8 Hz, 4H, iPr C$\underline{\text{H}}$), 3.53 (q, J = 6.8 Hz, 4H, Cu(OC$\underline{\text{H}}_2$CH$_3$)$_2$Mg), 2.89 (h, J = 7.0 Hz, 4H, iPr C$\underline{\text{H}}$), 2.65 (t, J = 5.8 Hz, NC$\underline{\text{H}}_2$CH$_2$), 1.75 (s, 6H, NC(C$\underline{\text{H}}_3$)CH), 1.40 - 1.35 (m, 2H, NCH$_2$C$\underline{\text{H}}_2$, overlaps with 1.33), 1.33 (d, J = 6.7 Hz, 12H, iPr C$\underline{\text{H}}_3$), 1.24 (dd, J = 7.0 Hz, 24H, iPr C$\underline{\text{H}}_3$), 1.10 (d, J = 6.9 Hz, 12H, iPr C$\underline{\text{H}}_3$), 0.79 (apparent t, J = 6.8 Hz, 6H, Cu(OCH$_2$C$\underline{\text{H}}_3$)$_2$Mg).

^{13}C{^{1}H} NMR (125 MHz, C$_6$D$_6$): δ 202.7 ($\underline{\text{C}}$Cu), 167.2 (N$\underline{\text{C}}$(CH$_3$)CH), 148.2 (Ar$\underline{\text{C}}$), 145.3 (Ar$\underline{\text{C}}$), 143.1 (Ar$\underline{\text{C}}$), 142.5 (Ar$\underline{\text{C}}$), 129.4 (Ar$\underline{\text{C}}$), 125.2 (Ar$\underline{\text{C}}$), 124.1 (Ar$\underline{\text{C}}$), 123.4 (Ar$\underline{\text{C}}$), 94.6 (NC(CH$_3$)$\underline{\text{C}}$H), 60.6 (Cu(O$\underline{\text{C}}$H$_2$CH$_3$)$_2$Mg), 47.4 (N$\underline{\text{C}}$H$_2$CH$_2$), 28.7 (iPr $\underline{\text{C}}$H), 28.0 (iPr $\underline{\text{C}}$H), 25.4 (iPr $\underline{\text{C}}$H$_3$), 25.2 (iPr $\underline{\text{C}}$H$_3$), 25.1 (iPr $\underline{\text{C}}$H$_3$), 24.6 (iPr $\underline{\text{C}}$H$_3$), 24.4 (NC($\underline{\text{C}}$H$_3$)CH), 23.9 (Cu(OCH$_2\underline{\text{C}}$H$_3$)$_2$Mg), 20.3 (NCH$_2\underline{\text{C}}$H$_2$).

Anal. Calc. for C$_{61}$H$_{91}$CuMgN$_4$O$_2$: C, 73.25; H, 9.17; N, 5.60 %. Found: C, 72.94; H, 9.03; N, 5.59 %.

[(6-Dipp)CuMgL] (2)

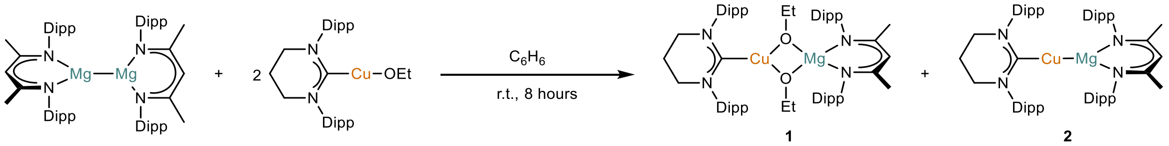

1
Yield: 99%

2
Yield: 34%

In a Schlenk flask to (LMg)$_2$ (660.0 mg, 1.0 eq, 746.7 µmol) and (6-Dipp)CuOEt (790 mg, 2.1 eq, 1.54 mmol) was added benzene (15 mL) and the resultant slurry stirred. After 2 hours additional (6-Dipp)CuOEt (76.7 mg, 0.2 eq, 149.3 µmol) was added and stirring was continued. After two further hours of stirring the volatiles were removed. The resulting crude solid was triturated with pentane (2 × 15 mL). The product was extracted twice with pentane and volatiles removed *in vacuo*. The resulting orange solid is then purified *via* recrystallisation from pentane (229.3 mg, 34 %). Suitable crystals for SCXRD were grown from a saturated solution in pentane at −30 °C.

^{1}H NMR (500 MHz, C$_6$D$_6$): δ 7.19 (t, *J* = 7.8 Hz, 2H, Ar$\underline{\text{H}}$), 7.13 – 7.10 (m, 4H, Ar$\underline{\text{H}}$), 7.0 (d, *J* = 7.8 Hz, 4H, Ar$\underline{\text{H}}$), 4.90 (s, 1H, NC(CH$_3$)C$\underline{\text{H}}$), 3.13 (h, *J* = 6.9 Hz, 4H, iPr C$\underline{\text{H}}$), 2.89 (h, *J* = 6.9 Hz, 4H, iPr C$\underline{\text{H}}$), 2.55 (t, *J*

= 5.9 Hz, 4H, NCH_2CH_2), 1.69 (s, 6H, $NC(CH_3)CH$), 1.40 (p, J = 6.2 Hz, 2H, NCH_2CH_2), 1.21 (m, 36H, iPr CH_3), 1.16 (d, J = 6.9 Hz, 12H, iPr CH_3).

$^{13}C\{^1H\}$ NMR (125 MHz, C_6D_6): δ 202.0 (CCu), 165.9 ($NC(CH_3)CH$), 147.0 (ArC), 145.3 (ArC), 141.8 (ArC), 141.5 (ArC), 128.8 (ArC), 128.6 (ArC), 124.4 (ArC), 124.3 (ArC), 123.5 (ArC), 94.6 ($NC(CH_3)CH$), 47.0 (NCH_2CH_2), 28.7 (iPr CH), 28.3 (iPr CH), 25.8 (iPr CH_3), 25.8 (iPr CH_3), 24.6 (iPr CH_3), 24.4 (iPr CH_3), 24.0 ($NC(CH_3)CH$), 20.7 (NCH_2CH_2).

IR (cm^{-1}) (ATR): 3067, 3031, 2967, 2873, 1523, 1438, 1400, 1319, 1104, 935.2, 846.3, 796.7, 756.2, 377.2.

Anal. Calc. for $C_{57}H_{81}CuMgN_4$: C, 75.22; H, 8.97; N, 6.16 %. Found: C, 74.99; H, 9.42; N, 5.57 %.

[(6-Dipp)Cu{μ-H}$_2$MgL] (3)

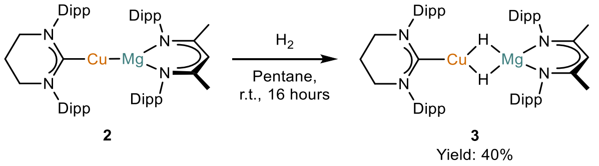

In a J. Young's ampoule **2** (100.0 mg, 109.9 μmol) was dissolved in pentane (20 mL). The resulting orange solution was degassed before an atmosphere of H_2 was added. Overnight the solution gradually became yellow. The product was filtered, and the solvent was concentrated to ca. 10 mL. The saturated pentane solution was placed stored at −30 °C which over the course of 48 hours resulted in crystals that were isolated (40.3 mg, 40 %). Suitable crystals for SCXRD were grown from a saturated solution in pentane at −30 °C. Despite repeated attempts, acceptable elemental analysis could not be obtained. This is unsurprising as **3** shows limited stability, in solution it slowly converts to [(6-Dipp)CuH]$_2$ and [LMgH]$_2$. Best attempt: $C_{57}H_{83}CuMgN_4$: C, 75.05; H, 9.17; N, 6.14 %. Found: C, 73.48; H, 8.58, N, 5.27 %.

^1H NMR (500 MHz, C_6D_6): δ 7.15 – 7.11 (m, 4H, ArH), 7.06 (d, J = 7.9 Hz, 4H, ArH), 6.93 (d, J = 7.7 Hz, 4H, ArH), 4.88 (s, 1H, $NC(CH_3)CH$), 3.18 (h, J = 6.9 Hz, 4H, iPr CH), 3.07 (h, J = 6.8 Hz, 4H, iPr CH), 2.74 (t, J = 5.9 Hz, 4H, NCH_2CH_2), 2.47 (s, 2H, CuH_2Mg), 1.63, (s, 6H, $NC(CH_3)CH$), 1.52 (p, J = 6.9 Hz 2 H, NCH_2CH_2), 1.24 (d, J = 6.8 Hz, 12H, iPr CH_3), 1.20 (d, J = 6.9 Hz, 12H, iPr CH_3), 1.16 (d, J = 6.9 Hz, 12H, iPr CH_3), 1.13 (d, J = 6.9 Hz, 12H, iPr CH_3).

$^{13}C\{^1H\}$ NMR (125 MHz, C_6D_6): δ 213.0 (CCu), 168.5 ($NC(CH_3)CH$), 145.6 (ArC), 145.5 (ArC), 142.5 (ArC), 142.1 (ArC), 128.8 (ArC), 128.6 (ArC), 124.7 (ArC), 124.0 (ArC), 94.1 ($NC(CH_3)CH$), 47.3 (NCH_2CH_2), 28.6 (iPr CH) 28.3 (iPr CH), 25.6 (iPr CH_3), 25.5 (iPr CH_3), 25.2 (iPr CH_3), 24.4 (iPr CH_3), 24.1 ($NC(CH_3)CH$), 20.8 (NCH_2CH_2).

IR (cm^{-1}) (ATR): 3070, 3032, 2967, 2875, 1438, 1402, 1317, 1178, 1105, 934.3, **901.7** (Cu{μ-H}$_2$Mg), 795.6, 757.8, **627.9** (Cu{μ-H}$_2$Mg), 390.4.

For the corresponding deuteride, **3*** ([(6-Dipp)Cu{μ-D}$_2$MgL]), synthesised via repetition of the above with D_2 gas.

IR (cm^{-1}) (ATR): 3068, 2967, 2875, 1408, 1319, 1178, 1105, 938.2, 796.1, 757.6, **701.1** (Cu{μ-D}$_2$Mg), **500.6** (Cu{μ-D}$_2$Mg), 393.3.

[(6-Dipp)CuC(NCy)$_2$MgL] (4)

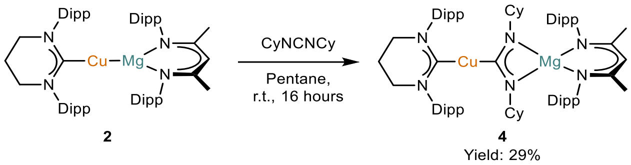

Compound **2** (80.0 mg, 1.0 eq 87.9 μmol) was dissolved in hexane (5 mL) and to it *N,N'*-dicyclohexylcarbodiimide (18.1 mg, 15.73 μL, 1.0 eq, 87.9 μmol) was added. This was allowed to stir overnight. The resulting solution was then filtered and placed in the freezer after which clear crystals were

isolated (28.5 mg, 29 %). Single crystals suitable for SCXRD were grown from a saturated hexane solution at −30 °C.

^{1}H NMR (500 MHz, C_6D_6): δ 7.27 – 7.17 (m, 6H, ArH), 7.09 (d, *J* = 7.7 Hz, 1H, ArH), 7.05 – 7.01 (m, 7H, ArH), 4.94 (s, 1H, NC(CH_3)CH), 3.58 (h, *J* = 6.7 Hz, 4H, iPr CH), 3.01 – 2.88 (m, 4H, iPr CH), 2.58 (t, *J* = 5.9 Hz, 4H, NCH_2CH_2), 1.76 (s, 6H, NC(CH_3)CH), 1.44 (d, *J* = 6.9 Hz, 12H, iPr CH_3), 1.36 (d, *J* = 6.7 Hz, 12H, iPr CH_3), 1.33 (d, *J* = 6.8 Hz, 12H, iPr CH_3), 1.18 (d, *J* = 7.0 Hz, 12H, iPr CH_3). Proton environments for the cyclohexyl regions were not assigned due to broadening and overlap with the iPr CH_3 environments.

^{13}C{^{1}H} NMR (125 MHz, C_6D_6): δ 215.1 (N_2CCu), 204.0 (CCu), 169.3 (NC(CH_3)CH), 146.8 (ArC), 145.9 (ArC), 145.1 (ArC), 125.2 (ArC), 124.9 (ArC), 124.6 (ArC), 124.0 (ArC), 123.8 (ArC), 94.6 (NC(CH_3)CH), 47.5 (NCH_2CH_2), 36.9 ((Cy-CH)N), 28.9 (iPr CH), 26.2 (iPr CH), 25.2 (iPr CH_3), 24.7 (iPr CH_3), 24.5 (iPr CH_3), 24.1 (NC(CH_3)CH), 22.7 (Cy-C) 20.5 (NCH_2CH_2).

MS (ESI) Expected: 1114.7391, found: 1115.7338 $[M+H]^+$ (err [ppm] = −3.45).

[(6-Dipp)CuSiMe$_2$Ph] (5)
Authentic synthesis adapted from the synthesis [(IMes)CuSiMe$_2$Ph].[4] For the *in-situ* formation *via* compound **2** see **NMR Scale Reactions**.

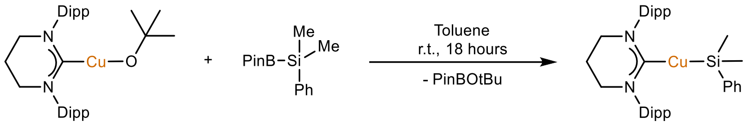

5
Yield: 78%

To a stirring suspension of (6-Dipp)CuOtBu (0.60 g, 1.0 eq. 1.1 mmol) in toluene (10 mL) was added a solution of PinBSiMe$_2$Ph (0.34 mL, 1.1 eq, 1.2 mmol) in toluene (30 mL). The resulting dark brown solution was stirred for 18 h, shielded from light. The reaction mixture was filtered, and volatiles were removed *in vacuo* to give solid brown residues. The crude material was washed with hexane (20 mL) and dried *in vacuo* to give 0.44 g of compound **5**. The hexane filtrate was stored at −30 °C overnight, resulting in the crystallisation and isolation of a further 0.08 g of compound **5** as a pale brown crystalline solid (total yield: 0.52 g, 78 %). Single crystals suitable for SCXRD were grown from benzene.

^{1}H NMR (500 MHz, C_6D_6): δ 7.34 – 7.28 (m, 2H, ArH), 7.27 – 7.18 (m, 5H, ArH), 7.09 (d, *J* = 7.8 Hz, 4H, ArH), 2.99 (h, *J* = 6.9 Hz, 4H, iPr CH), 2.68 (t, *J* = 5.9 Hz, 4H, NCH_2CH_2), 1.47 (p, *J* = 6.5 Hz, 2H, NCH_2CH_2), 1.40 (d, *J* = 6.9 Hz, 12H, iPr CH_3), 1.18 (d, *J* = 6.9 Hz, 12H, iPr CH_3), 0.27 (s, 6H, SiCH_3).

^{13}C{^{1}H} NMR (125 MHz, C_6D_6): δ 203.8 (CCu), 153.5 (ArC), 145.9 (ArC), 141.3 (ArC), 135.2 (ArC), 129.3 (ArC), 126.9 (ArC), 125.7 (ArC), 124.7 (ArC), 45.8 (NCH_2CH_2), 28.9 (iPr CH), 25.1 (iPr CH_3), 24.7 (iPr CH_3), 20.4 (NCH_2CH_2), 4.3 (SiCH_3).

^{29}Si NMR (99 MHz, C_6D_6): δ −14.3 (SiMe$_2$Ph).

Anal. Calc. for $C_{36}H_{51}CuN_2Si$: C, 71.65; H, 8.52; N, 4.64 %. Found: C, 71.31; H, 8.48; N, 4.64 %.

[(6-Dipp)CuFeCp(CO)$_2$] (6)
Authentic synthesis adapted from the synthesis of (IPr)CuFe(CO)$_2$Cp.[5] For the *in-situ* formation *via* compound **2** see **NMR Scale Reactions**.

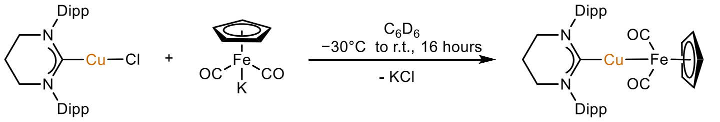

2 **6**
Yield: 13%

(6-Dipp)CuCl (157.6 mg, 1.0 eq, 312.9 µmol) was dissolved in THF (20 mL). Separately, KFe(CO)$_2$Cp (67.6 mg, 1.0 eq, 312.9 µmol) was dissolved in THF (20 mL) and the light brown solution cooled to −78 °C *via* an acetone/dry ice bath. The yellow THF solution containing (6-Dipp)CuCl (157.6 mg, 1 eq, 312.9 µmol) was then added dropwise to the KFe(CO)$_2$Cp solution. The resultant brown solution was stirred overnight, whilst

warming to room temperature. The brown solution was pumped down *in vacuo* to dryness, with the brown solid obtained subsequently washed 3 times with hexane (3 × 15 mL). The remaining brown solid was dissolved in toluene (15 mL) and filtered. A layer of hexane (10 mL) was then added to the orange/red solution, which was cooled to −30 °C overnight. Yellow crystals were isolated and dried *in vacuo* (25.4 mg, 13 %).

^{1}H NMR (400 MHz, C_6D_6): δ 7.25 – 7.19 (m, 2H, ArH), 7.14 (d, *J* = 7.4 Hz, 4H, ArH), 4.04 (s, 5H, CpH), 3.11 (h, *J* = 7.0 Hz, 4 H, iPr CH), 2.78 (t, *J* = 6.0 Hz, 4H, NCH_2CH_2), 1.61 (d, *J* = 6.9 Hz, 12H, iPr CH_3), 1.52 (p, *J* = 6.0 Hz, 2H, NCH_2CH_2), 1.20 (d, *J* = 6.9 Hz, 12H, iPr CH_3).

$^{13}C\{^1H\}$ NMR (100 MHz, C_6D_6): δ 220.6 (FeCO), 198.2 (CCu), 145.7 (ArC), 142.2 (ArC), 129.6 (ArC), 125.1 (ArC), 77.5 (Cp C), 46.5 (NCH_2CH_2), 29.0 (iPr CH), 25.1 (iPr CH_3), 24.6 (iPr CH_3), 20.3 (NCH_2CH_2).

Anal. Calc. for $C_{35}H_{45}CuFeN_2O_2$: C, 65.16; H, 7.03; N, 4.34 %. Found: C, 65.08; H, 7.02; N, 4.49 %.

[(6-Dipp)CuZnL] (7)

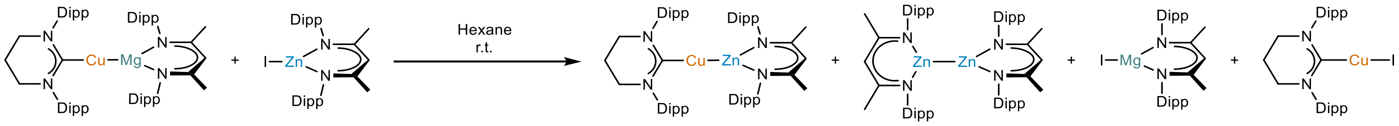

2 7 Yield: 10%

Compound **2** (100.0 mg, 1.0 eq 109.9 µmol) was dissolved in hexane (5 mL). Over the course of 48 hours, LZnI (67.0 mg, 1.0 eq, 109.9 µmol) was added in 0.25 eq increments. This was allowed to stir for a further 48 hours. The resulting orange suspension was filtered and concentrated to dryness *in vacuo*. The product was recrystallised from pentane, yielding orange crystals (10.0 mg, 10 %) a small impurity was attributed to compound **2** which co-crystalised. Crystals suitable for SCXRD experiment were grown from a C_6D_6/pentane mixture at −35 °C.

^{1}H NMR (400 MHz, C_6D_6): δ 7.20 (d, *J* = 7.8 Hz, 2H, ArH), 7.14 – 7.10 (m, 4H, ArH), 6.98 (d, *J* = 7.8 Hz, 4H, ArH), 4.94 (s, 1H, $NC(CH_3)CH$), 3.16 (h, *J* = 6.9 Hz, 4H, iPr CH), 2.83 (h, *J* = 7.0 Hz, 4H, iPr CH), 2.51 (t, *J* = 5.7 Hz, 4H, NCH_2CH_2), 1.70 (s, 6H, $NC(CH_3)CH$), 1.35 (p, *J* = 5.7 Hz, 2H, NCH_2CH_2), 1.23 (d, *J* = 7.0 Hz, 12H, iPr CH_3), 1.18 (d, *J* = 7.0 Hz, 12H, iPr CH_3), 1.15 (d, *J* = 6.9 Hz, 12H, iPr CH_3), 1.10 (d, *J* = 6.9 Hz, 12H, iPr CH_3).

$^{13}C\{^1H\}$ NMR (100 MHz, C_6D_6): δ 203.0 (CCu), 163.6, ($NC(CH_3)CH$), 149.0 (ArC), 145.1 (ArC), 141.7 (ArC), 141.4 (ArC), 129.1 (ArC), 124.7 (ArC), 123.7 (ArC), 123.4 (ArC), 94.4 ($NC(CH_3)CH$), 47.3 (NCH_2CH_2), 28.8 (iPr CH), 28.3 (iPr CH), 26.0 (iPr CH_3), 25.8 (iPr CH_3), 24.5 ($NC(CH_3)CH$), 24.3 (iPr CH_3), 23.7 (iPr CH_3), 20.7 (NCH_2CH_2).

Anal. Calc. for $C_{57}H_{81}CuN_4Zn$: C, 71.97; H, 8.58; N, 5.89 %. Found: C, 72.53; H, 8.45; N, 5.28 %.

ICP-OES on sample of **7** exposed to air and dissolved in nitric acid, average of two repeats: Cu, 4.95; Zn, 4.36 % m/m. Zn:Cu molar ratio, 0.86:1.

NMR Scale Reactions

[(6-Dipp)CuMgL] (2) + ^{t}BuOH

In a J. Young's NMR tube **2** (20.5 mg, 1.0 eq, 22.5 µmol) was dissolved in C_6D_6 (0.5 mL). To it ^{t}BuOH (2.15 µL, 1.67 mg, 1.0 eq, 22.5µmol) was added. After 30 minutes the orange-coloured solution faded to a yellow colour at which point the solution was probed by ^{1}H NMR spectroscopy. Another equivalent of ^{t}BuOH (2.15 µL, 1.67 mg, 1.0 eq, 22.5µmol) was added and the reaction was again probed by ^{1}H NMR spectroscopy, noting compound **2** was now completely consumed.

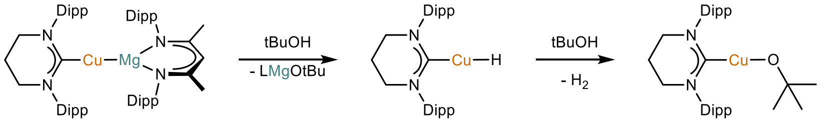

2

Whilst this reaction not selective, and two consecutive reactions take place simultaneously as show in the scheme above. The first is the direct reaction of [(6-Dipp)CuMgL] (**2**) with ^tBuOH which results in the formation of the (6-Dipp)CuH and LMgO^{t}Bu species, the former of which is readily seen in the ^1H NMR spectrum (S35). The newly generated (6-Dipp)CuH species can then react with ^tBuOH, releasing H_2 gas, observable by the singlet species at 4.47 ppm in the ^1H NMR spectrum, and (6-Dipp)CuO^{t}Bu also observable in the ^1H NMR spectrum. This consecutive reaction results in residual **2** which was completely consumed to provide principally [(6-Dipp)CuOtBu] and some residual [(6-Dipp)CuH] after adding additional ^tBuOH (see Fig. S35).

[(6-Dipp)CuMgL] (2) + HSi(Me)$_2$Ph

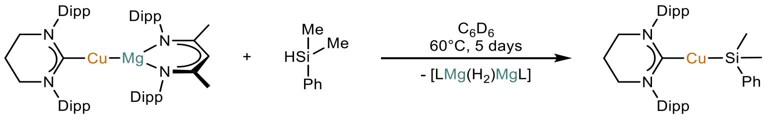

2 5

NMR Yield: 50%

In a J. Young's NMR tube **2** (14.0 mg, 1.0 eq, 15.4 μmol) was dissolved in C_6D_6 (0.5 mL). To it dimethyl(phenyl)silane (2.37 μL, 2.10 mg, 1.0 eq, 15.4 μmol) was added. The solution was heated to 60 °C for 5 days after which **2** was no longer present in the NMR spectrum and the product, [(6-Dipp)CuSiMe$_2$Ph] **(5)** was identified. 1,3,5-Trimethoxybenzene (9.6 mg) was added as a calibrant to determine the NMR yield of **5** (4.65 mg, 50 %).

Spectroscopic data matched that of compound **5** as synthesised above.

[(6-Dipp)CuMgL] (2) + PhCl

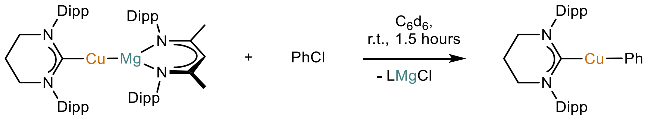

2

NMR Yield: 82%

In a J. Young's NMR tube **2** (19.5 mg, 1.0 eq, 21.4 μmol) was dissolved in C_6D_6 (0.5 mL). To it chlorobenzene (2.18 μL, 2.41 mg, 1.0 eq, 21.4 μmol) was added. The solution was allowed to react over the course of 1.5 hours. ^1H NMR showed complete consumption of **2** and a quantitative conversion to (6-Dipp)CuPh. 1,3,5-Trimethoxybenzene (10.9 mg) was added as a calibrant to determine the NMR yield (7.11 mg, 82 %). Additional peaks are attributed to LMgCl side product.

Spectroscopic data matched that of (6-Dipp)CuPh in the literature [3].

[(6-Dipp)CuMgL] (2) + Ph$_2$POMe

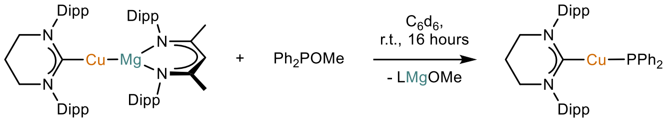

2

NMR Yield >99%

In a J. Young's NMR tube **2** (14.87 mg, 1.0 eq. 16.34 μmol) was dissolved in C_6D_6 (0.5 mL). To it methoxy(diphenyl)phosphane (3.27 μL, 3.53 mg, 1.0 eq, 16.34 μmol) was added. The solution was allowed to react overnight. ^1H and ^{31}P NMR showed complete consumption of the phosphine and generation of the product, (6-Dipp)CuPPh$_2$. 1,3,5-Trimethoxybenzene (13.8 mg) was added as a calibrant to determine the NMR yield (> 99%).

Spectroscopic data matched that of (6-Dipp)CuPPh$_2$ in the literature.[6]

[(6-Dipp)CuMgL] (2) + CpFe(CO)$_2$I

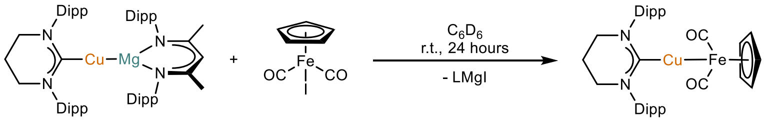

2 6

NMR Yield: 51%

In a J. Young's NMR tube **2** (20.0 mg, 1.0 eq, 22.0 µmol) was dissolved in C_6D_6 (0.5 mL). To it $CpFe(CO)_2I$ (6.7 mg, 1.0 eq, 22.0 µmol) was added. This addition rapidly led to the dissolution of remaining **2** and darkening of the solution. Over a 24-hour period the solution became a lighter orange and brown and colourless material precipitated out. 1,3,5-Trimethoxybenzene (12.8 mg) was added as a calibrant to determine the NMR yield of **6** (7.3 mg, 51 %).

Spectroscopic data matched that of compound **6** as synthesised above.

Supplementary Note 2

In-situ monitoring of [(6-Dipp)CuMgL] (2) + LZnI

In order to account for the low yield of **7**, its synthesis from **2** and LZnI was monitored via NMR spectroscopy in the presence of a calibrant. In a J. Young's NMR tube **2** (12.7 mg, 1.0 eq, 14.0 μmol) was dissolved in C_6D_6 (0.5 mL). To it LZnI (8.51 mg, 1.0 eq, 14.0 μmol) was added. The reaction was left for 16 hours at RT. The calibrant, 1,3,5-trimethoxybenzene (12.1 mg, 71.9 μmol) was added to the solution to determine the NMR yields of the products.

Supplementary Table 1 **Data from spectrum in Supplementary Fig. 33.** From the integration of the $(OMe)_3$ proton environment the number of moles each normalised integral represents can be determined.

Mass (g)	Molecular Mass /g mol^{-1}	Moles /μmol	Integral of $(Me)_3$ groups	Actual Protons	Normalised Integral	Moles/Normalised Integral /μmol
0.0121	168.19	71.9	9	9	1	71.9

With ratio of moles to the normalised integral of the $(OMe)_3$ nine protons, the moles, and hence the mass of any other NMR observable species can be calculated from the equation below.

$$(\text{Normalised integral of compound}) \times (71.9\ \mu\text{mol}) = \text{Moles of compound x in solution}$$

Supplementary Table 2 **The calculated masses of the major species present in Supplementary Fig. 33.** The lower mass of LMgI was attributed to poor solubility.

Compound	Integral	Actual Protons	Normalised Integral	Calculated Moles /μmol	Calculated Mass /mg
[(6-Dipp)CuZnL]	0.06	1	0.060	4.32	4.1
[(6-Dipp]CuMgL]	0.05	1	0.050	3.60	3.3
LZnZnL	0.11	2	0.055	3.96	3.8
6DippCuI	0.31	4	0.078	5.58	3.3
LMgI	0.07	2	0.035	2.52	1.4
			Total	20.0	16.0

Thus, of an expected 21.2 mg, 28 μmol of compounds present in solution 20 μmol could be directly observed. Of the 14 μmol of **2** added, 13.5 μmol could be traced to diamagnetic, 6-Dipp containing compounds The mass of LMgI was notably low, due to its poor solubility and likely constitutes the remaining unobserved mass of 5.2 mg.

To provide evidence that **7** was reacting with LZnI to generate LZnZnL and [(6-Dipp)CuI] More LZnI (5.7 mg,0.67 eq, 8.8 μmol) was added and allowed to react for a further 16 hours at RT. This resulted in a decrease in intensity of the resonance attributed to **7**, and enhancement of intensity of the resonances associated with LZnZnL and [(6-Dipp)CuI]

Supplementary Table 3 **The integrals of species present in Supplementary Fig. 34 before and after an additional amount of LZnI was added.**

Compound	1 eq. LZnI	2 eq. LZnI
[(6-Dipp)CuZnL]	0.06	0.040
[(6-Dipp]CuMgL]	0.05	0.020
LZnZnL	0.11	0.210
6DippCuI	0.31	0.430
LMgI	0.07	0.090

Supplementary Note 3

Data for **3**, **4** and **6** were collected on an Agilent Supernova diffractometer (using Cu-Kα radiation), **7** was collected on a Synergy diffractometer (using Cu-Kα radiation), while those for **1**, **2**, **5** and **[(6-Dipp)CuOEt]** were obtained using an Agilent Xcalibur instrument and a Mo-Kα source. All experiments were conducted at 150 K. Structures were solved using SHELXT [7] and refined using SHELXL[8] via the Olex-2[9] interface. Where disorder prevailed, distance and ADP restraints were employed, on merit, to assist convergence. Additional noteworthy points follow.

The bridging ethoxy carbon atoms were treated for 82:18 disorder in the structure of **1**. All hydrogen atoms were included at calculated positions with the exception of those attached to C58 and C60 (82% occupancy). These latter four were located and each refined at a distance of 0.98 Å from the relevant parent atom.

In **2**, the asymmetric unit was seen to contain one molecule of the bimetallic complex and a region of solvent. There were three areas of disorder in the main feature, namely, C3 (85:15), the methyl groups attached to C11 (60:40) and the entire isopropyl moiety based on C52 (50:50). All disorders were readily modelled and the refinement converged well. The solvent void was identifiable as being shared between an equal proportion of benzene and pentane. However, the electron density therein indicated substantial smearing and, hence, this region of the electron density map was ultimately treated using the solvent mask available in Olex-2. Allowance has been made for the solvent presence in the formula as presented.

In addition to one molecule of the complex, the asymmetric unit in **3** plays host to a molecule of pentane. C3 in the main feature was modelled to take account of 80:20 disorder while the methyl groups in the isopropyl moiety based on C55 were subject to 65:35 disorder. Bridging hydride ligands were located and refined without restraints. The solvent also fell prey to disorder which was readily modelled as a 50:50 split.

The asymmetric unit in **4** contains two molecules of the bimetallic complex and two molecules of hexane. Disorder (60:40), in the molecule based on Cu1, was modelled for C2, C3 and C4 as well as the methyl groups attached to C11 and C23 and the entire isopropyl functionalities containing on C26 and C65. In addition, 50:50 disorder was accommodated for the cyclohexyl ligand bound to N10 and the dipp group bonded to N11, in the complex based on Cu2. While the solvent was broadly identifiable, smearing of the electron-density for one of these moieties was manifested to an extent that a disorder model would have been over-parameterized. As such, the solvent was ultimately addressed via the solvent-mask algorithm available in Olex-2.

The largest residual electron density maximum, in the structure of **6**, is at a chemically insignificant distance from the copper centre. Attempts made to model this peak as copper disorder were ultimately abandoned as the refined occupancy of the putative minor component was in the region of 2%, thus indicating electron-density that is approximately equivalent to a hydrogen atom.

The asymmetric unit in **7** comprises one molecule of the bimetallic complex and one molecule of pentane. C3 was modelled for 85:15 disorder, while the pentane was treated for 55:45 disorder with the inclusion of appropriate ADP and distance restraints. In another life, we might have decided to squeeze the solvent. However, with the goal of obtaining some meaningful, parallel refinements in relation to the metal positions, our aim was to model everything possible so that the effective (but perhaps more variable) solvent-mask algorithm would not skew results.

Our findings are as follows:

(i) Copper coordinated to carbene and zinc to NacNac: R1 = 0.0262, wR2 = 0.0739.

(ii) Copper coordinated to NacNac and zinc to carbene: R1 = 0.0306, wR2 = 0.0833.

(iii) Copper coordinated to both carbene and NacNac: R1 = 0.0269, wR2 = 0.0744

(iv) Zinc coordinated to both carbene and NacNac: R1 = 0.0289, wR2 = 0.0833.

In summary, the crystallographic preference is for Cu1 to be coordinated to carbene and Zn1 to be NacNac bound (as presented herein). We appreciate that this preference is marginal, and given that other results

indicate the presence of 10% of a Cu/Cu complex, there may be some contamination of the latter in the crystal chosen. In presenting these findings, our aim has been to maximise scientific rigour, throughout.

The asymmetric unit in **[(6-Dipp)CuOEt]** plays host to a toluene moiety with half site-occupancy, plus one molecule of the copper complex. The solvent straddles a crystallographic inversion centre and, hence, is disordered with itself. Distance and ADP restraints have been used in this region, to assist convergence.

Crystallographic data for all compounds have been deposited with the Cambridge Crystallographic Data Centre as supplementary publications CCDC 2363357-2363362 for **1**, **2**, **3**, **4**, **6** and **7**, respectively, CCDC 2265494 for **5** and CCDC 2408960 for **[(6-Dipp)CuOEt]**. Copies of these data can be obtained free of charge on application to CCDC, 12 Union Road, Cambridge CB2 1EZ, UK [fax(+44) 1223 336033, e-mail: deposit@ccdc.cam.ac.uk.

Structure plots represent the copper containing species in each of **1**, **2**, **3**, **4**, **5**, **6**, **7** and **[(6-Dipp)CuOEt]**. Only one of the two molecules in the asymmetric unit of **4** is illustrated. Ellipsoids are shown at 30% probability. Hydrogens have been omitted for clarity in all cases, barring the hydrides in **3**. Solvent and minor disordered components have also been omitted and carbene substituents have been universally depicted as wireframes, also for visual ease.

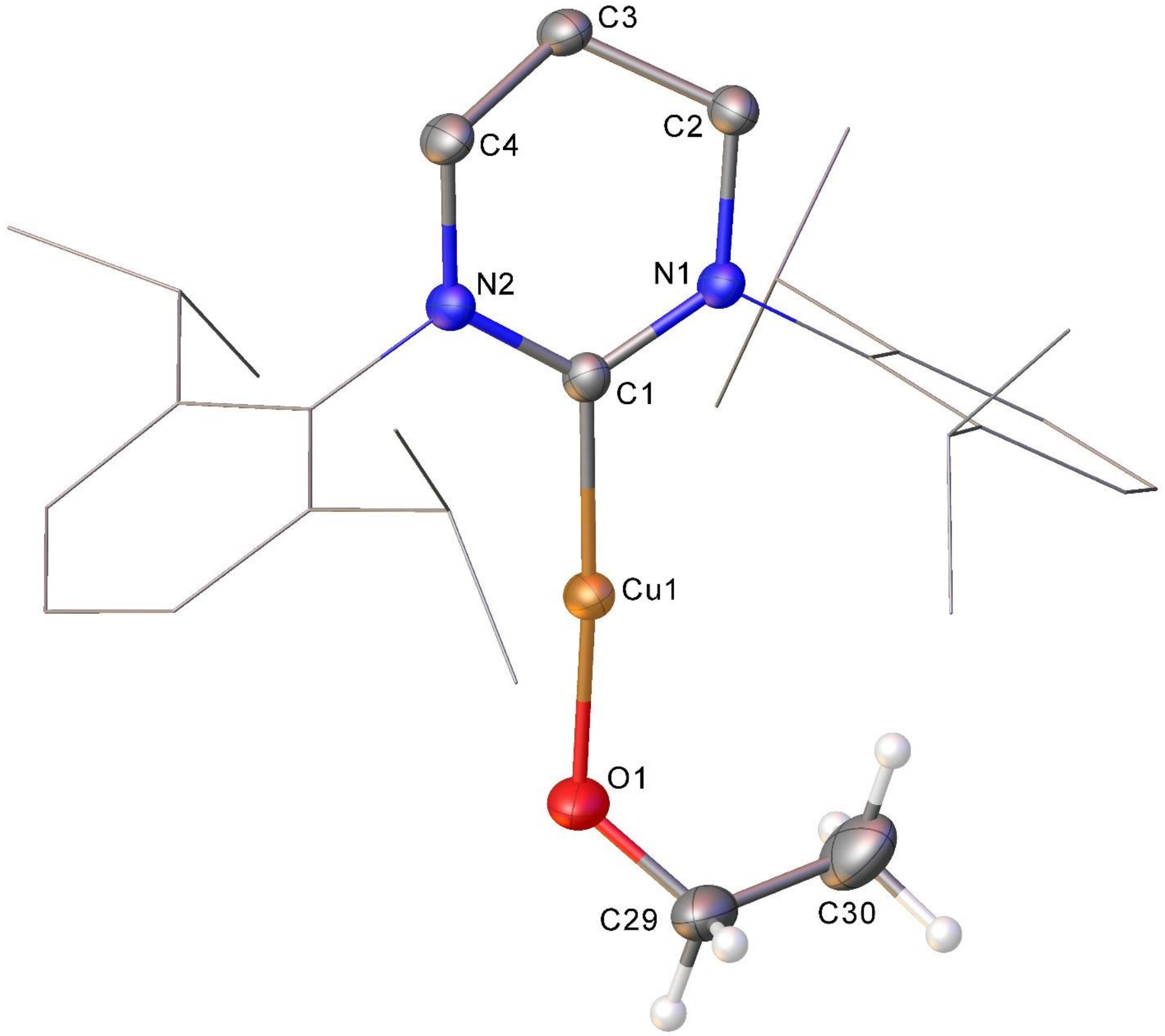

Supplementary Fig. 1 **Molecular structure of (6-Dipp)CuOEt.** Ellipsoids are shown at 30% probability. Hydrogen atoms have been omitted. Diisopropylphenyl groups have been represented in wireframe view, for clarity. CCDC: 2408960

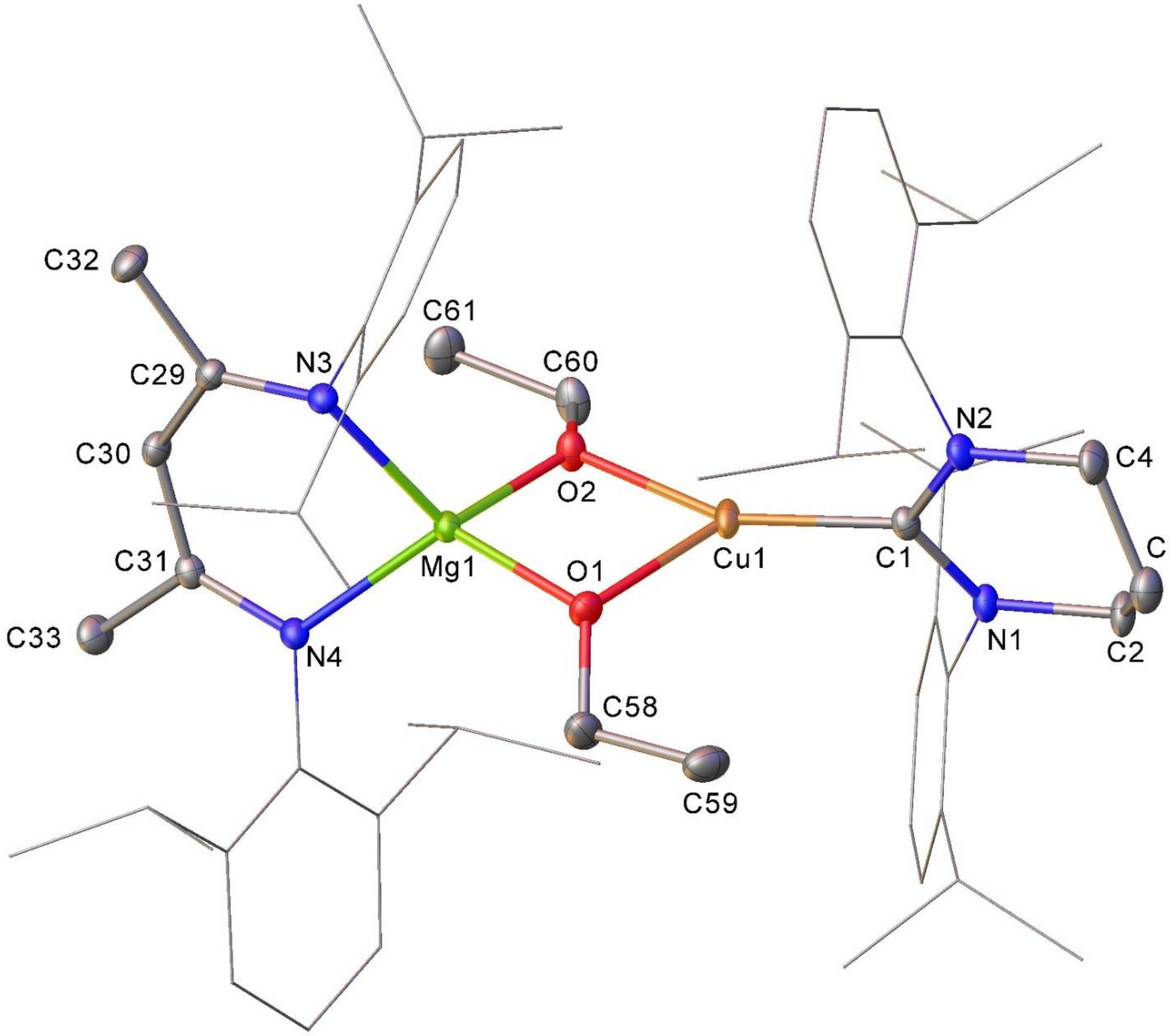

Supplementary Fig. 2 **Molecular structure of [(6-Dipp)Cu(μ_2-OEt)$_2$MgL] (1).** Ellipsoids are shown at 30% probability. Hydrogen atoms have been omitted. Diisopropylphenyl groups have been represented in wireframe view, for clarity. CCDC: 2363357

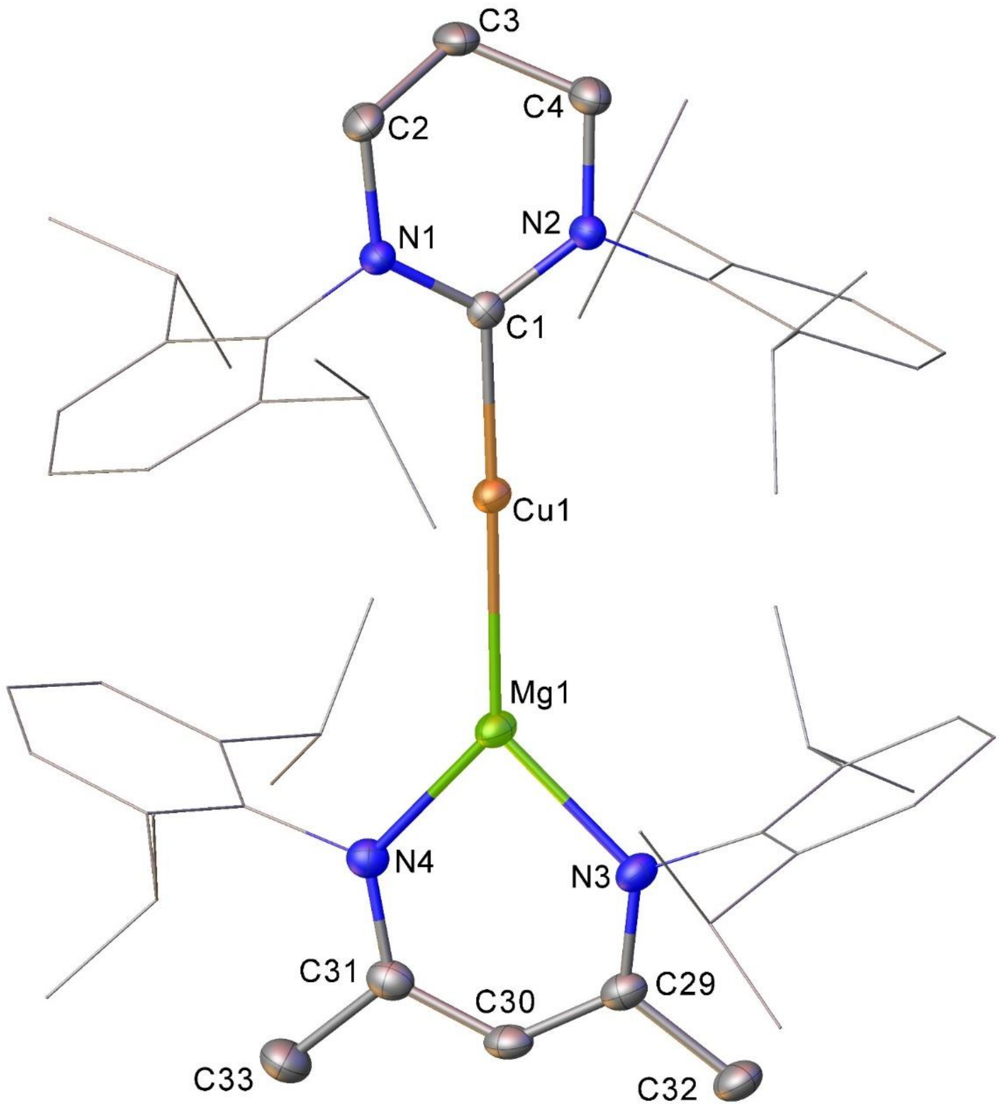

Supplementary Fig. 3 **Molecular structure of [(6-Dipp)CuMgL] (2).** Ellipsoids are shown at 30% probability. Hydrogen atoms have been omitted. Diisopropylphenyl groups have been represented in wireframe view, for clarity. CCDC: 2363358

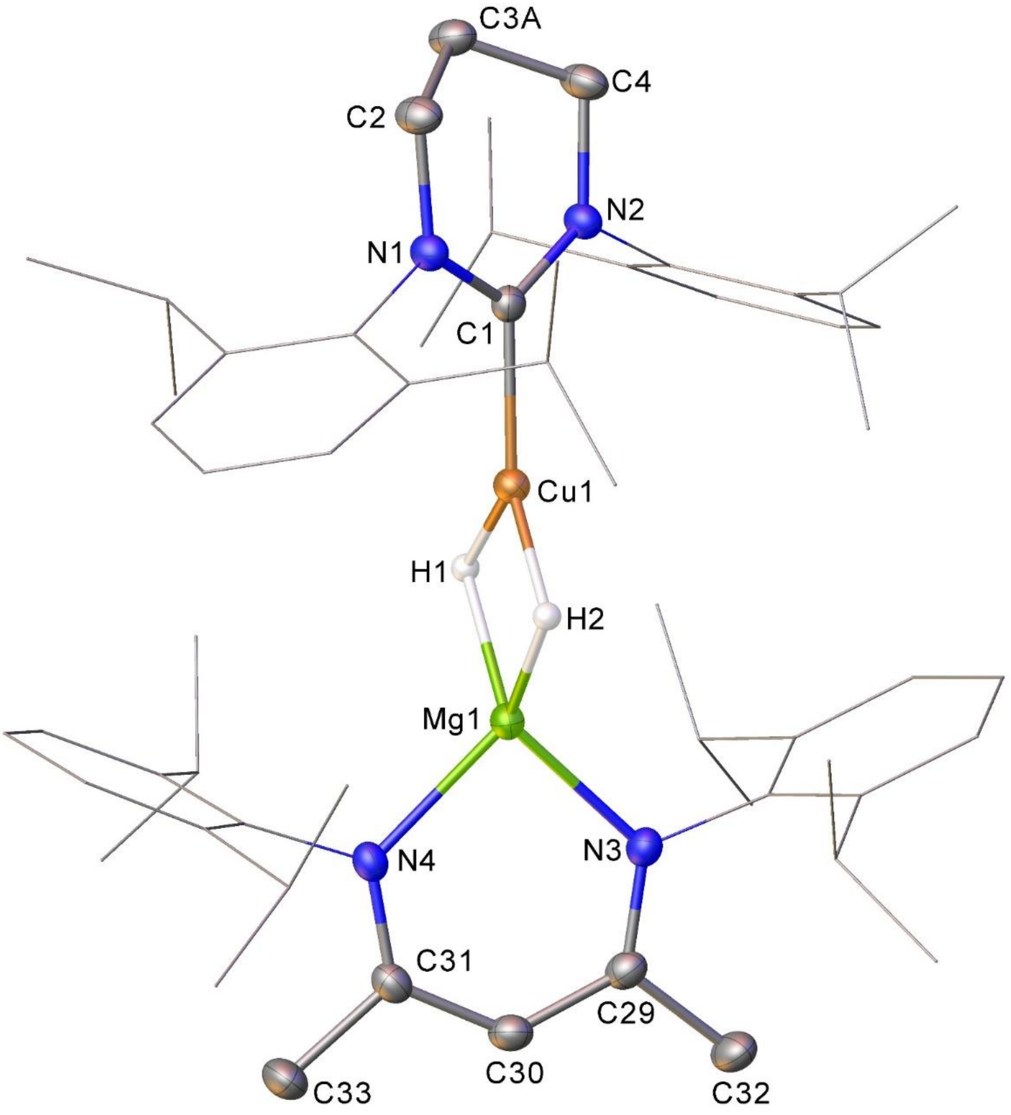

Supplementary Fig. 4 **Molecular structure of [(6-Dipp)Cu{μ-H}$_2$MgL] (3).** Ellipsoids are shown at 30% probability. Hydrogen atoms have been omitted. Diisopropylphenyl groups have been represented in wireframe view, for clarity. CCDC: 2363359

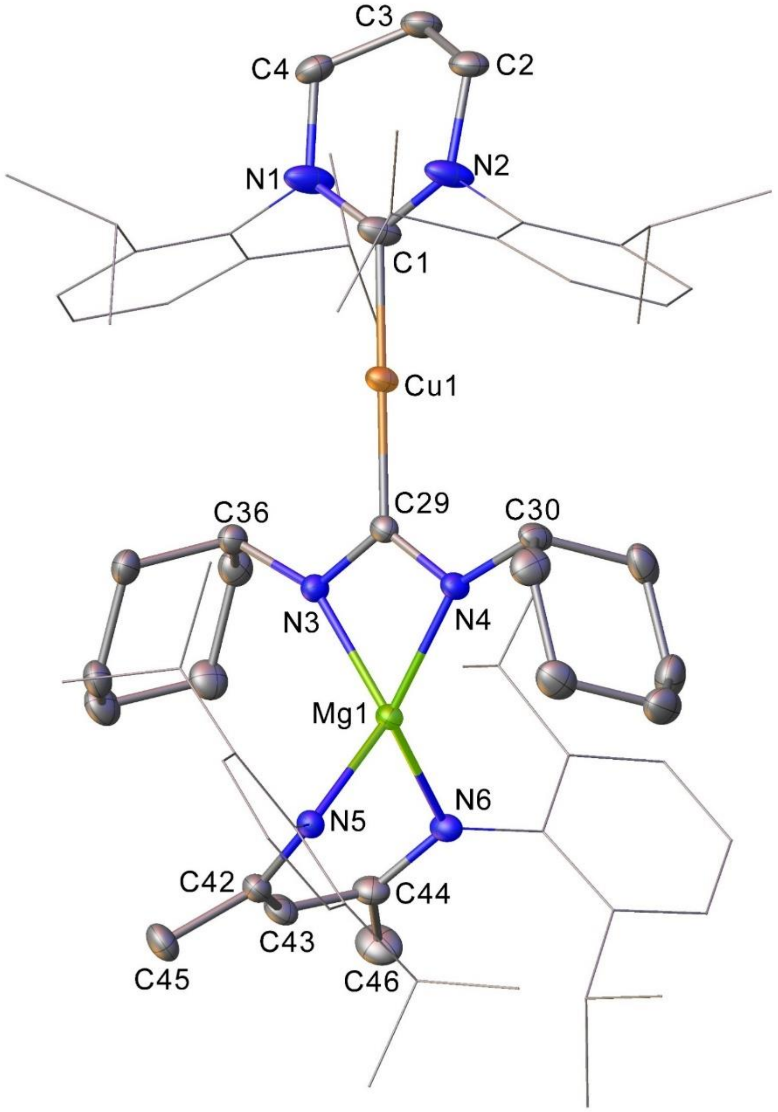

Supplementary Fig. 5 **Molecular structure of [(6-Dipp)CuC(NCy)$_2$MgL] (4).** Ellipsoids are shown at 30% probability. Hydrogen atoms have been omitted. Diisopropylphenyl groups have been represented in wireframe view, for clarity. CCDC: 2363360

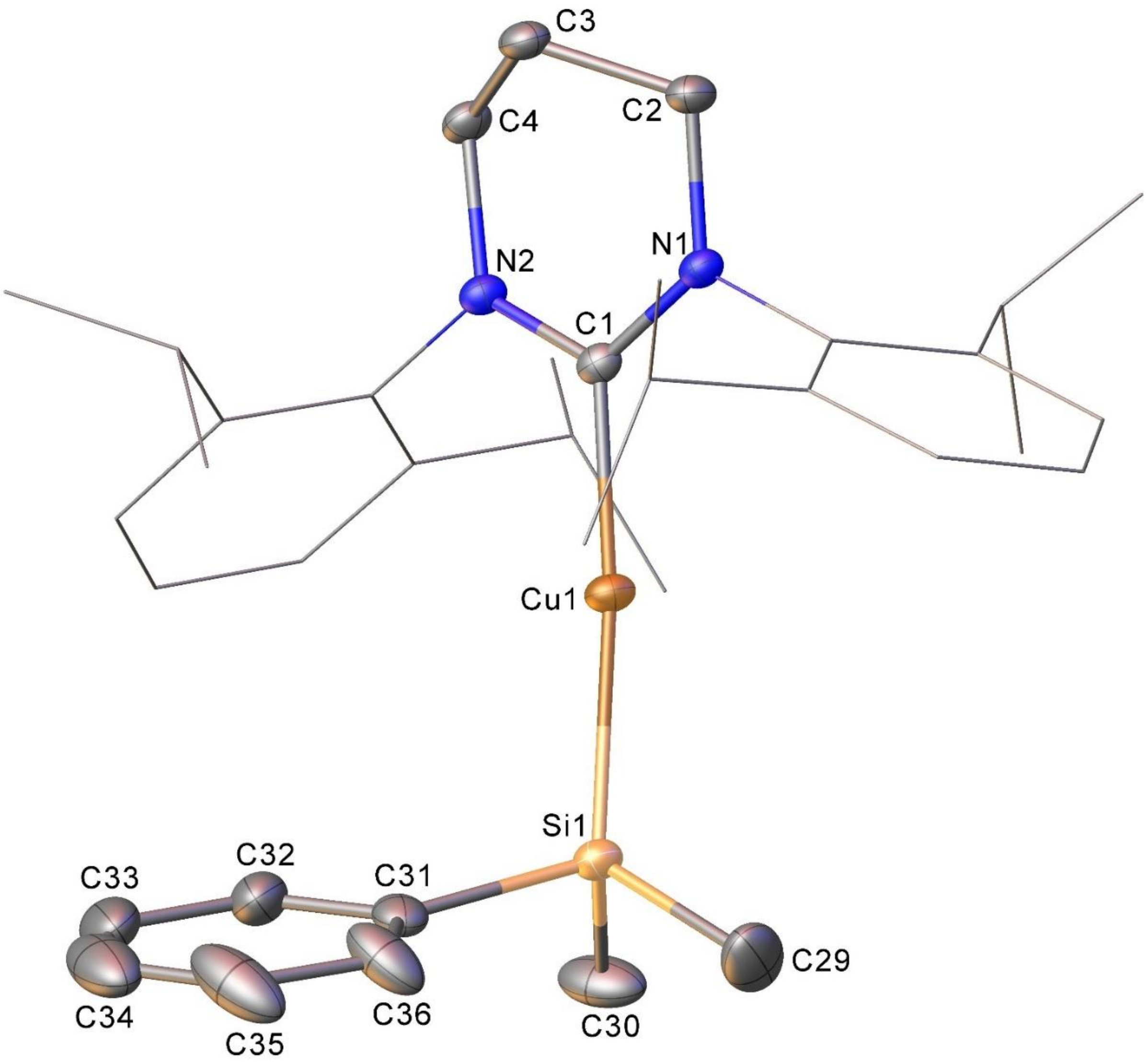

Supplementary Fig. 6 **Molecular structure [(6-Dipp)CuSiMe$_2$Ph] (5).** Ellipsoids are shown at 30% probability. Hydrogen atoms have been omitted. Diisopropylphenyl groups have been represented in wireframe view, for clarity. CCDC: 2265494

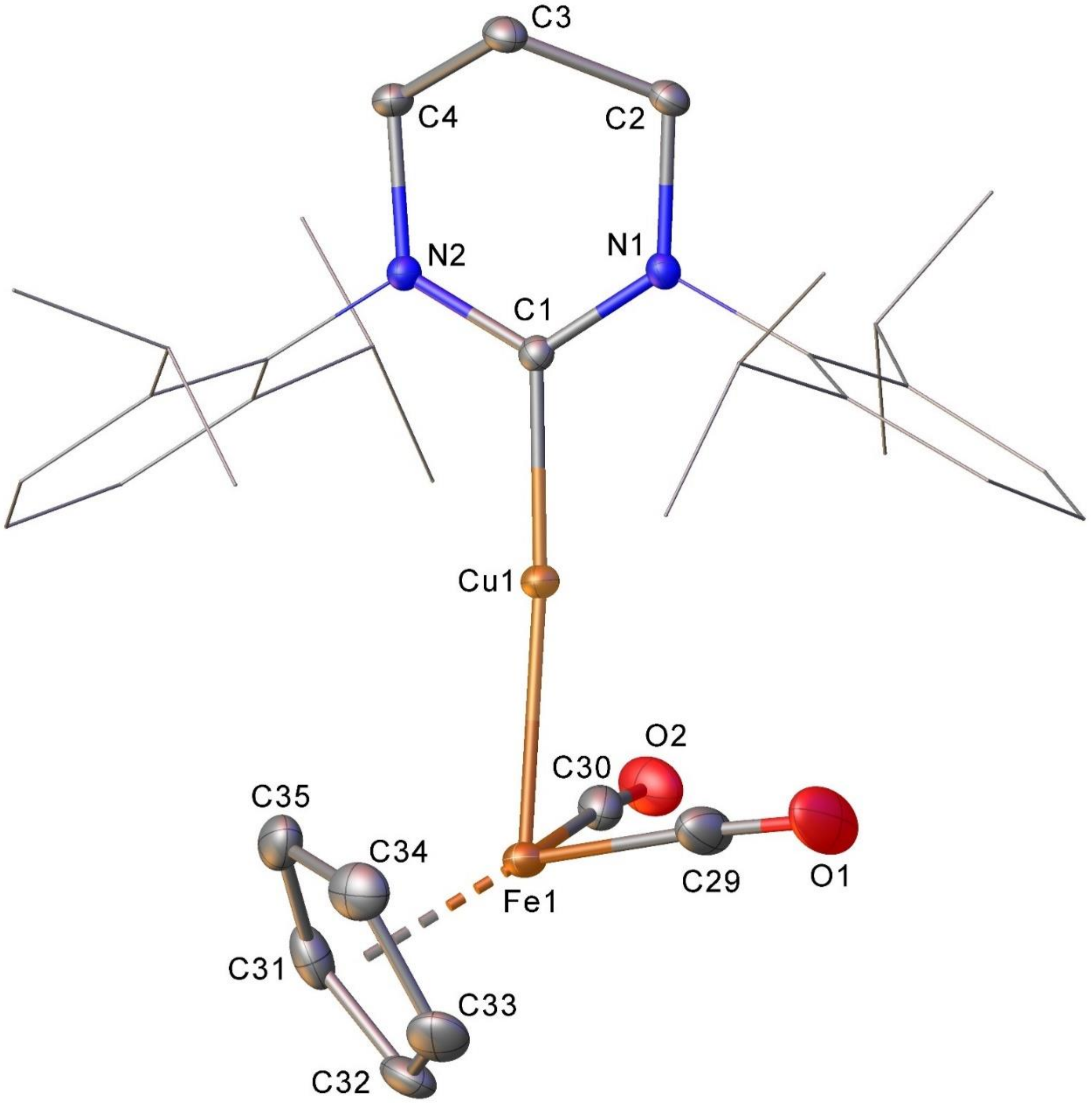

Supplementary Fig. 7 **Molecular structure [(6-Dipp)CuFeCp(CO)$_2$] (6).** Ellipsoids are shown at 30% probability. Hydrogen atoms have been omitted. Diisopropylphenyl groups have been represented in wireframe view, for clarity. CCDC: 2363361

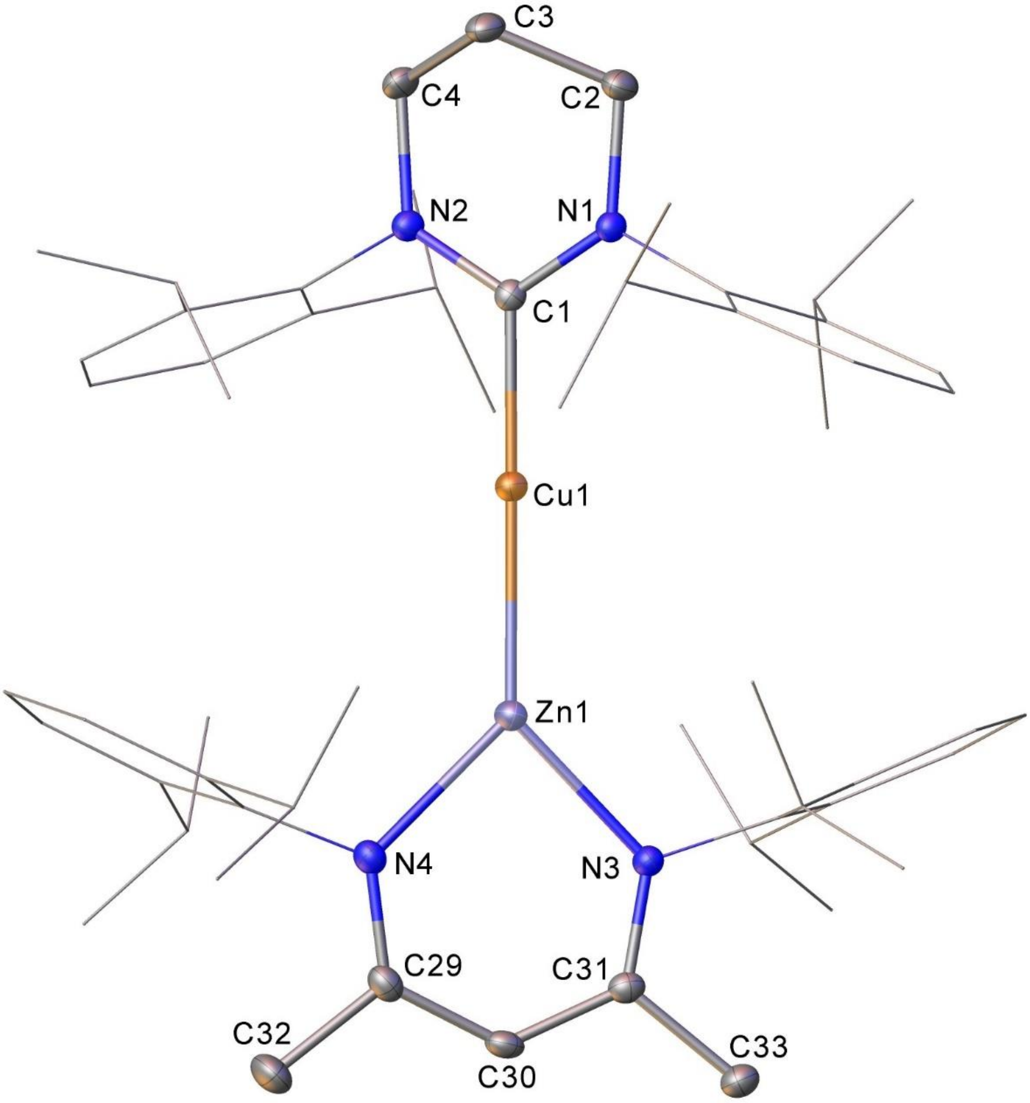

Supplementary Fig. 8 **Molecular structure [(6-Dipp)CuZnL] (7).** Ellipsoids are shown at 30% probability. Hydrogen atoms have been omitted. Diisopropylphenyl groups have been represented in wireframe view, for clarity. CCDC: 2363362

Supplementary Table 4 **Data collection and refinement details for 1, 2, 3 and 4.**

Manuscript Reference	**1**	**2**	**3**	**4**
Empirical formula	$C_{61}H_{91}CuMgN_4O_2$	$C_{62.5}H_{90}CuMgN_4$	$C_{62}H_{95}CuMgN_4$	$C_{152}H_{234}Cu_2Mg_2N_{12}$
Formula weight	1000.22	985.23	984.26	2405.20
Crystal system	monoclinic	orthorhombic	orthorhombic	triclinic
Space group	$P2_1/c$	$P2_12_12_1$	$P2_12_12_1$	P–1
a/ Å	17.5930(2)	14.1661(4)	14.3206(1)	12.2682(2)
b/ Å	14.8028(1)	17.7674(5)	17.9311(1)	22.7870(3)
c/ Å	22.5078(2)	23.4615(6)	23.2675(2)	26.1108(2)
α/ °	90	90	90	91.268(1)
β/ °	104.1250(10)	90	90	96.661(1)
γ/ °	90	90	90	91.785(1)
U/ Å^3	5684.39(9)	5905.1(3)	5974.72(7)	7244.19(16)
Z	4	4	4	2
ρ_{calc}/ g cm^{-3}	1.169	1.108	1.094	1.103
μ/ mm^{-1}	0.440	0.420	0.898	0.830
F(000)	2168.0	2136.0	2144.0	2624.0
Crystal size/ mm^3	0.569 × 0.405 × 0.329	0.524 × 0.239 × 0.207	0.17 × 0.128 × 0.106	0.232 × 0.207 × 0.123
2θ range for data collection/°	5.968 to 60.818	5.752 to 60.622	7.248 to 146.702	7.258 to 146.39
Index ranges	–24 ≤ h ≤ 23, –20 ≤ k ≤ 19, –29 ≤ l ≤ 31	–19 ≤ h ≤ 20, –22 ≤ k ≤ 24, –29 ≤ l ≤ 32	–17 ≤ h ≤ 17, –22 ≤ k ≤ 22, –25 ≤ l ≤ 28	–15 ≤ h ≤ 15, –28 ≤ k ≤ 26, –31 ≤ l ≤ 32
Reflections collected	54882	60316	81936	99870
Independent reflections, R_{int}	15106, 0.0289	15435, 0.0360	11946, 0.0348	28786, 0.0262
Data/restraints/parameters	15106/80/696	15435/121/644	11946/158/717	28786/839/1702
Goodness–of–fit on F^2	1.070	1.021	1.035	1.036
Final R1, wR2 [I>=2σ(I)]	0.0462, 0.1088	0.0411, 0.0854	0.0287, 0.0785	0.0382, 0.1041
Final R1, wR2 [all data]	0.0687, 0.1200	0.0629, 0.0932	0.0300, 0.0798	0.0438, 0.1085
Largest diff. peak/hole/ e Å^{-3}	0.92/–0.94	0.25/–0.30	0.38/–0.26	0.49/–0.52
Flack Parameter	–	–0.009(3)	–0.037(6)	–

Supplementary Table 5 **Data collection and refinement details for 5, 6, 7 and (6-Dipp)CuOEt.**

Manuscript Reference	**5**	**6**	**7**	**(6-Dipp)CuOEt**
Empirical formula	$C_{36}H_{51}CuN_2Si$	$C_{35}H_{45}CuFeN_2O_2$	$C_{62}H_{93}CuN_4Zn$	$C_{33.5}H_{49}CuN_2O$
Formula weight	603.41	645.12	1023.31	559.28
Crystal system	monoclinic	monoclinic	orthorhombic	monoclinic
Space group	$P2_1/n$	$P2_1$	$P2_12_12_1$	$P2_1/n$
a/ Å	13.3915(4)	8.7674(1)	14.3540(1)	12.36442(18)
b/ Å	16.0695(5)	16.9267(2)	17.6872(1)	21.1200(2)
c/ Å	16.3996(4)	11.1096(2)	23.2414(1)	12.37886(14)
α/ °	90	90	90	90
β/ °	91.806(2)	99.9920(10)	90	101.9006(13)
γ/ °	90	90	90	90
U/ Å^3	3527.35(17)	1623.69(4)	5900.58(6)	3163.09(7)
Z	4	2	4	4
ρ_{calc}/ g cm^{-3}	1.136	1.320	1.152	1.174
μ/ mm^{-1}	0.678	4.631	1.226	1.159
F(000)	1296.0	680.0	2208.0	1204.0
Crystal size/ mm^3	0.326 × 0.255 × 0.163	0.195 × 0.154 × 0.126	0.274 × 0.17 × 0.117	0.147 × 0.109 × 0.063
2θ range for data collection/°	5.58 to 60.582	8.08 to 145.702	6.28 to 160.618	8.374 to 146.4
Index ranges	$-18 \le h \le 17$, $-22 \le k \le 21$, $-22 \le l \le 21$	$-9 \le h \le 10$, $-20 \le k \le 20$, $-13 \le l \le 13$	$-18 \le h \le 18$, $-20 \le k \le 22$, $-29 \le l \le 27$	$-15 \le h \le 15$, $-26 \le k \le 25$, $-12 \le l \le 15$
Reflections collected	34771	13060	98210	27667
Independent reflections, R_{int}	9344, 0.0308	5862, 0.0248	12727, 0.0322	6239, 0.0639
Data/restraints/parameters	9344/0/371	5862/1/387	12727/160/689	6239/44/368
Goodness–of–fit on F^2	1.014	1.037	1.027	1.056
Final $R1$, $wR2$ [I>=2σ(I)]	0.0405, 0.0942	0.0305, 0.0786	0.0262, 0.0733	0.0475, 0.1275
Final $R1$, $wR2$ [all data]	0.0632, 0.1048	0.0311, 0.0792	0.0267, 0.0739	0.0537, 0.1347
Largest diff. peak/hole/ e Å^{-3}	0.48/–0.41	0.62/–0.29	0.29/-0.30	0.43/-0.95
Flack Parameter	–	–0.001(3)	-0.030(4)	-

Supplementary Figures

NMR Spectra

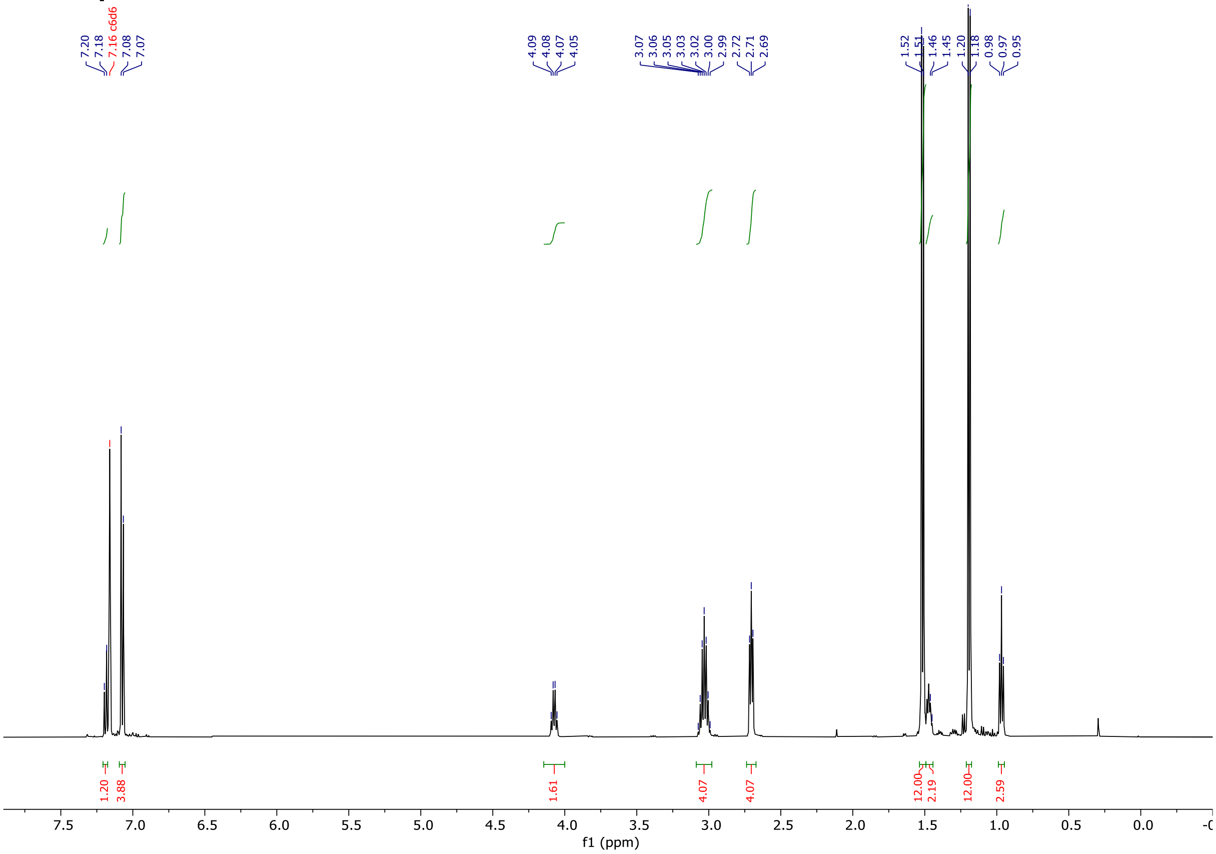

Supplementary Fig. 9 **The ^{1}H NMR spectrum (500 MHz, C_6D_6) of (6-Dipp)CuOEt.**

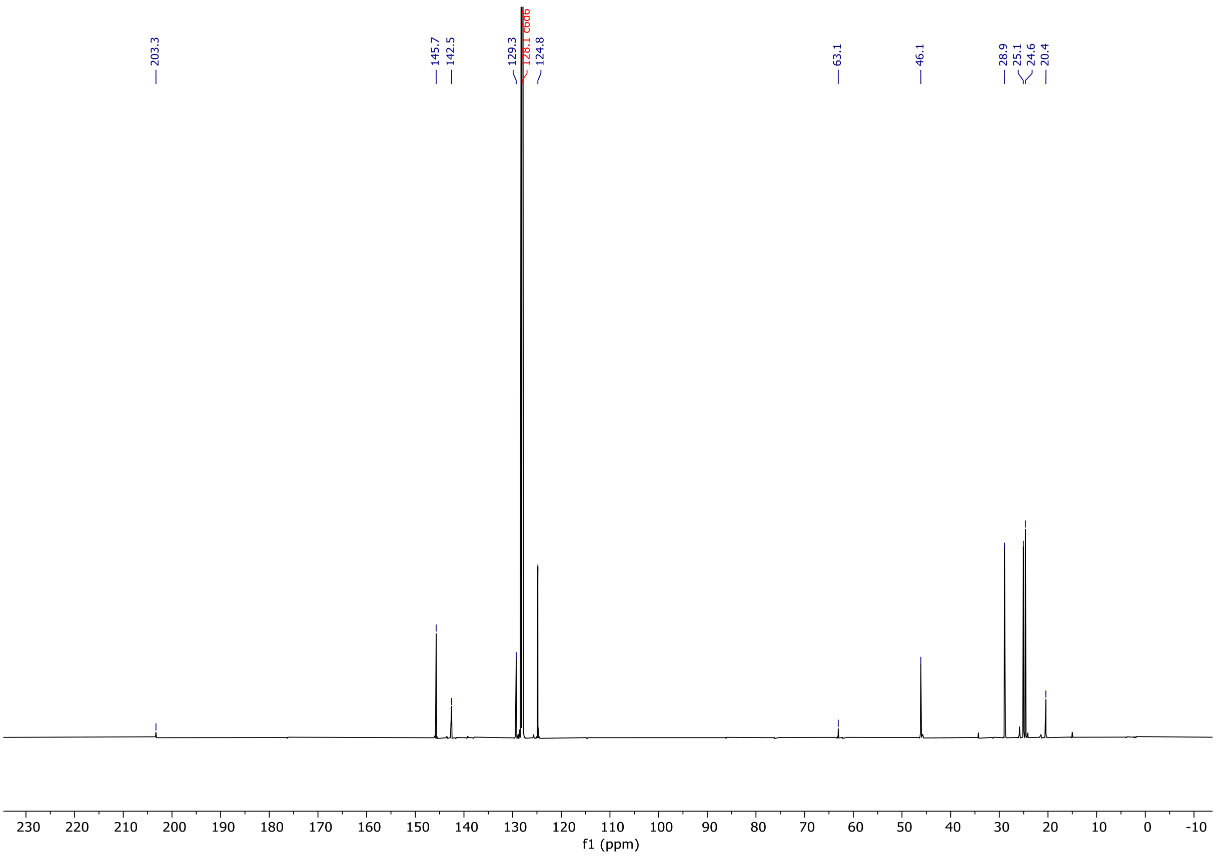

Supplementary Fig. 10 **The $^{13}C\{^{1}H\}$ NMR spectrum (125 MHz, C_6D_6) of (6-Dipp)CuOEt.**

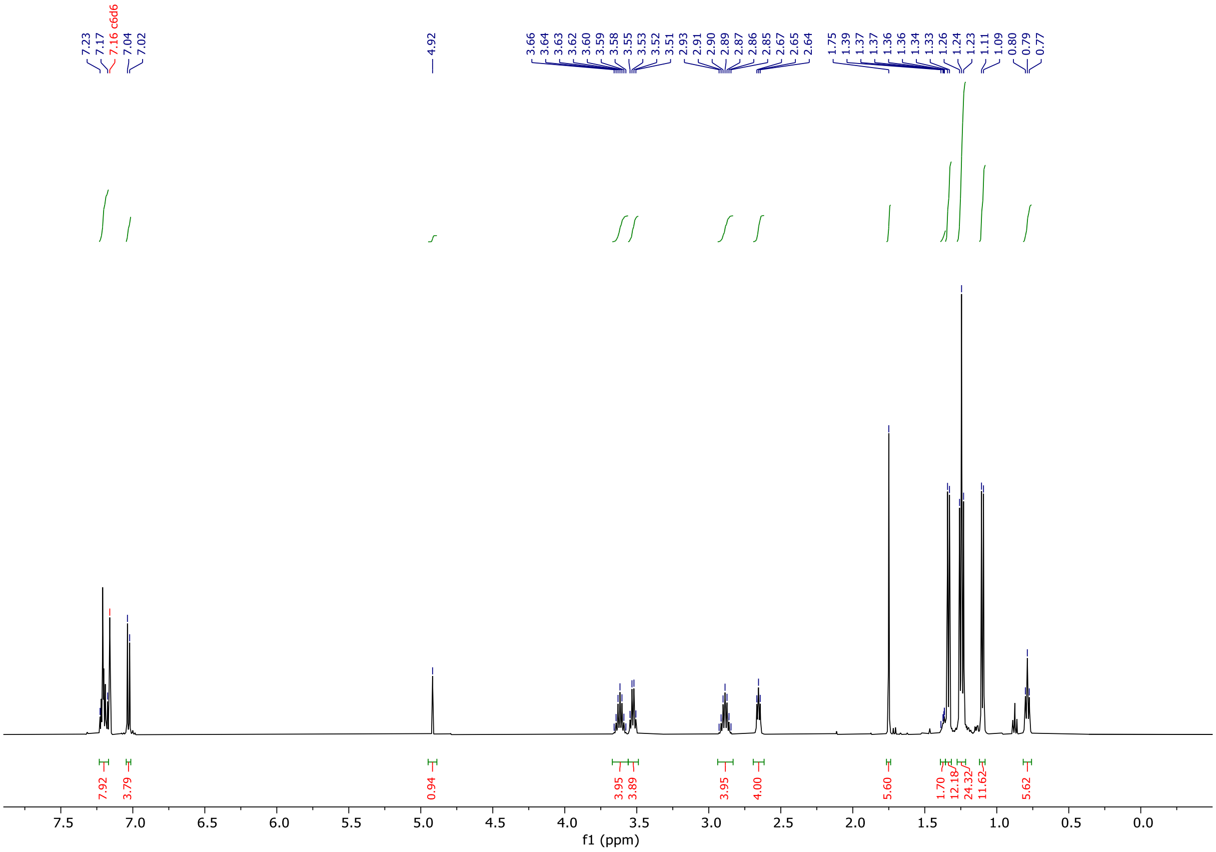

Supplementary Fig. 11 **The ^{1}H NMR spectrum (500 MHz, C_6D_6) of [(6-Dipp)Cu(μ_2-OEt)$_2$MgL] (1).**

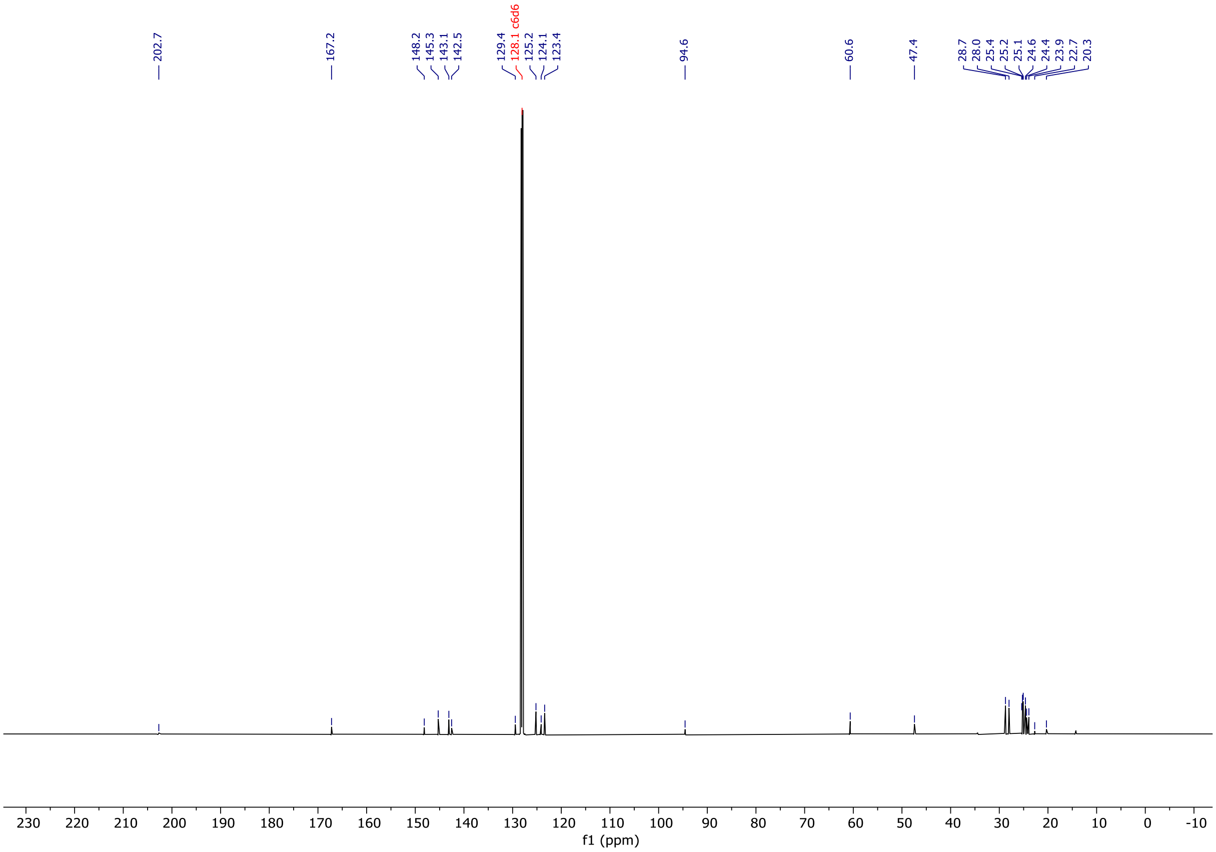

Supplementary Fig. 12 **The $^{13}C\{^{1}H\}$ NMR spectrum (125 MHz, C_6D_6) of [(6-Dipp)Cu(μ_2-OEt)$_2$MgL] (1).**

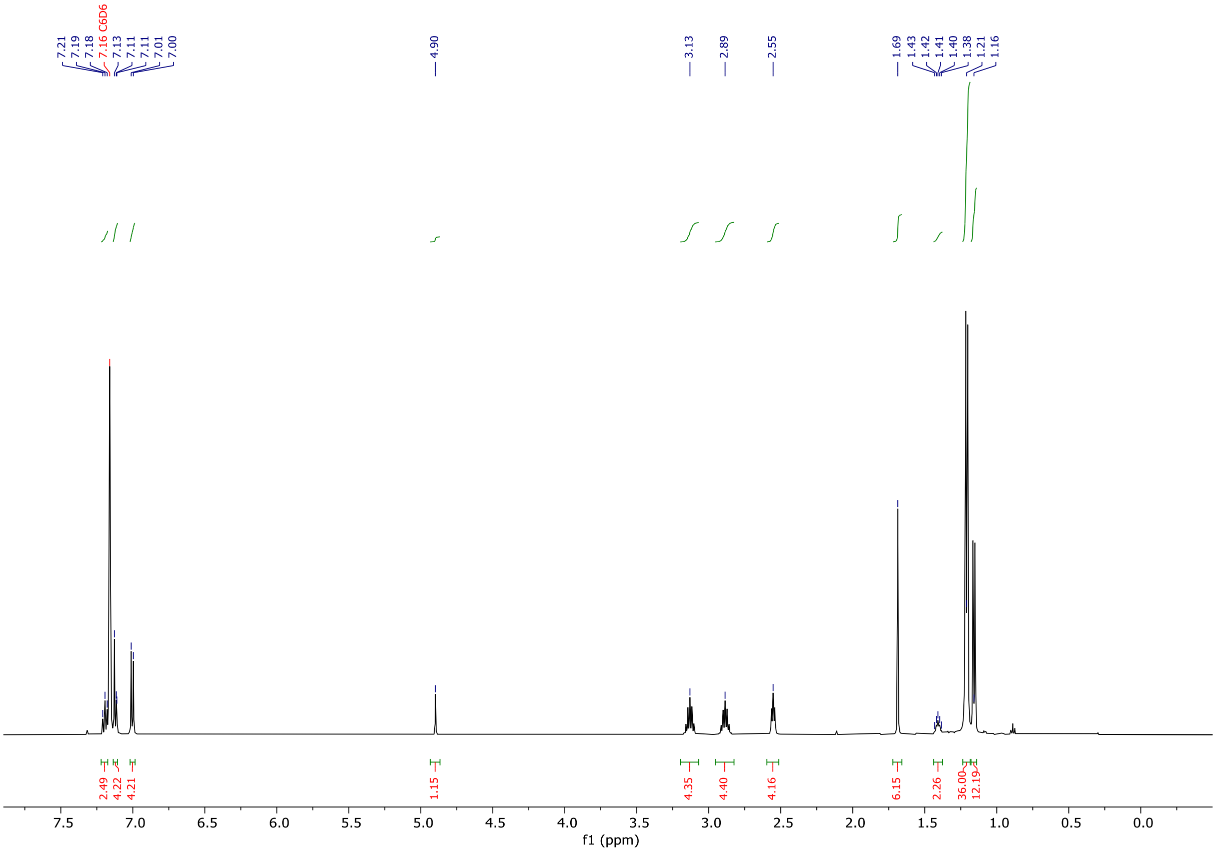

Supplementary Fig. 13 **The ^{1}H NMR spectrum (500 MHz, C_6D_6) of [(6-Dipp)CuMgL] (2).**

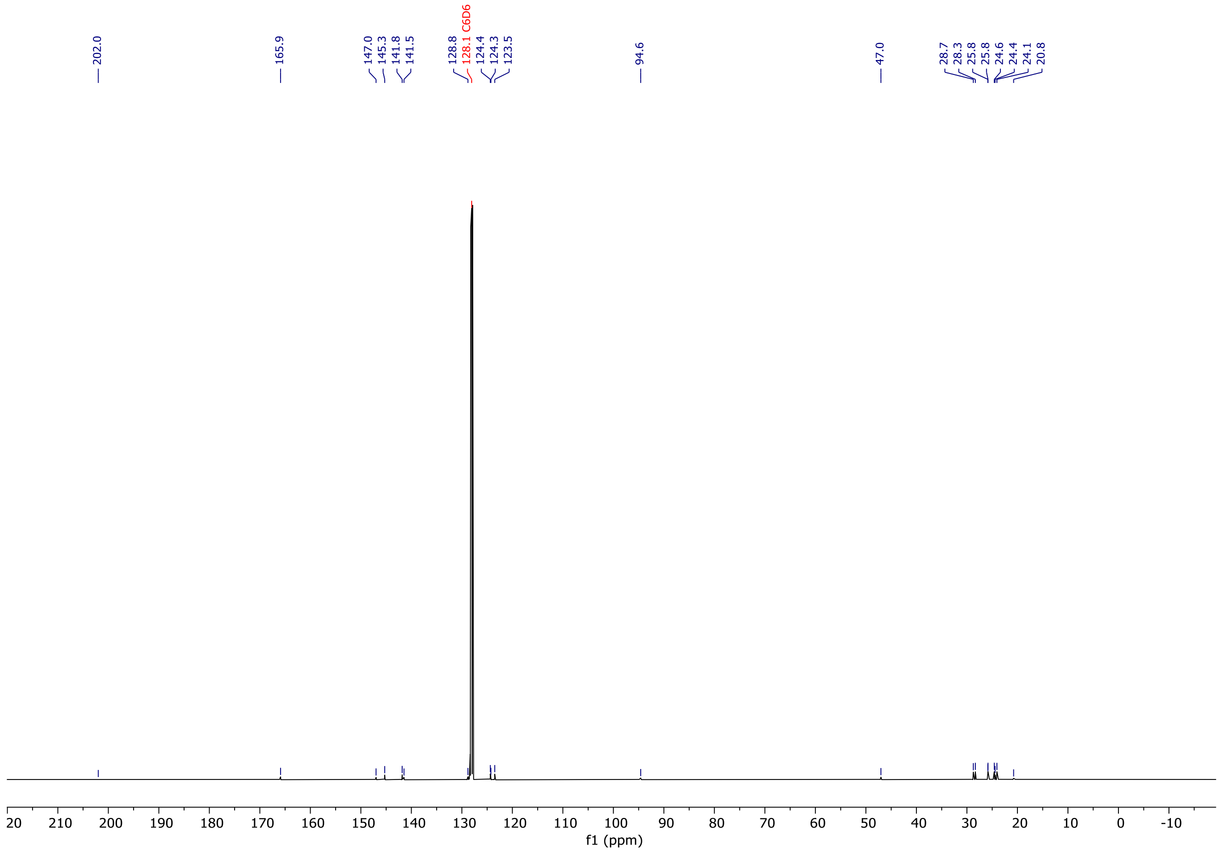

Supplementary Fig. 14 **The $^{13}C\{^{1}H\}$ NMR spectrum (125 MHz, C_6D_6) of [(6-Dipp)CuMgL] (2).**

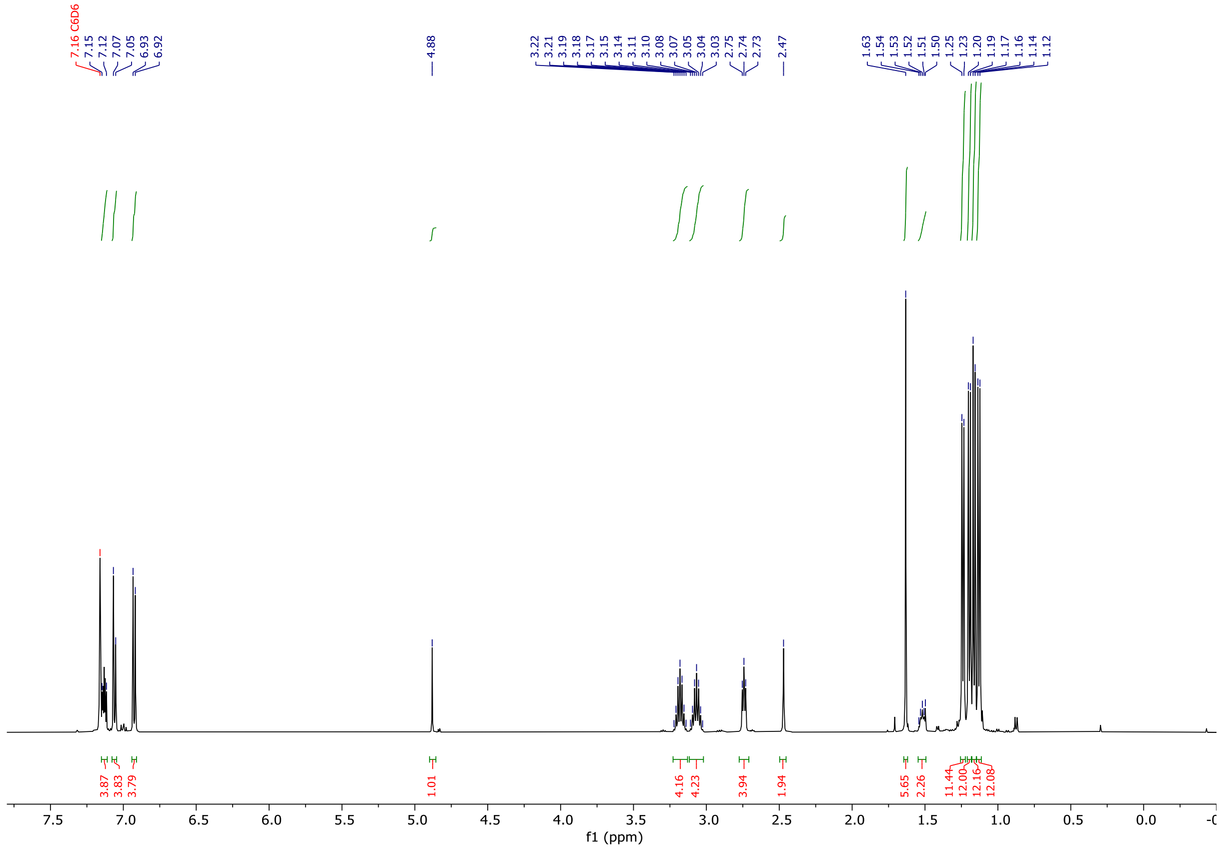

Supplementary Fig. 15 **The ^{1}H NMR spectrum (500 MHz, C_6D_6) of [(6-Dipp)Cu{μ-H}$_2$MgL] (3).**

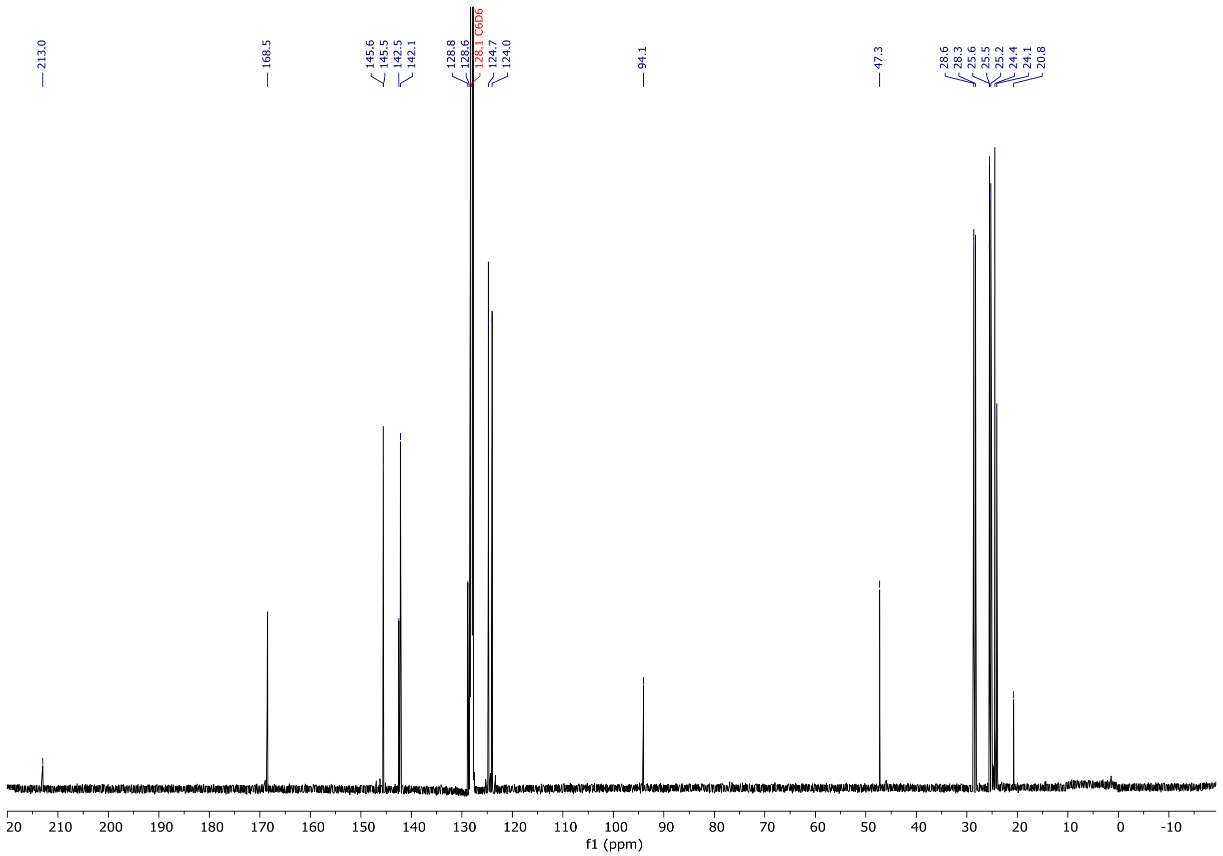

Supplementary Fig. 16 **The $^{13}C\{^{1}H\}$ NMR spectrum (125 MHz, C_6D_6) of [(6-Dipp)Cu{μ-H}$_2$MgL] (3).**

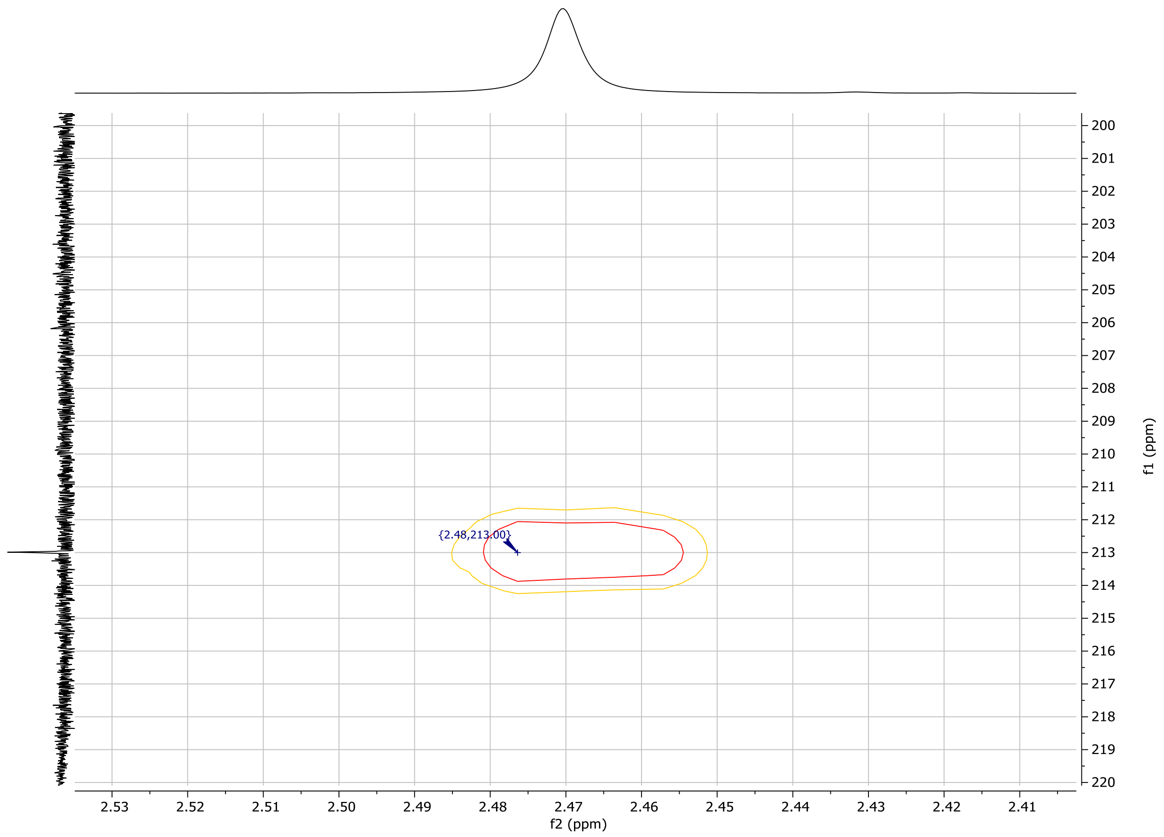

Supplementary Fig. 17 **HMBC (125 MHz, C_6D_6) of [(6-Dipp)Cu{μ-H}$_2$MgL] (3).** Displaying the $^{2}J_{H\text{-}C}$ coupling between the carbene carbon and the bridging hydrides.

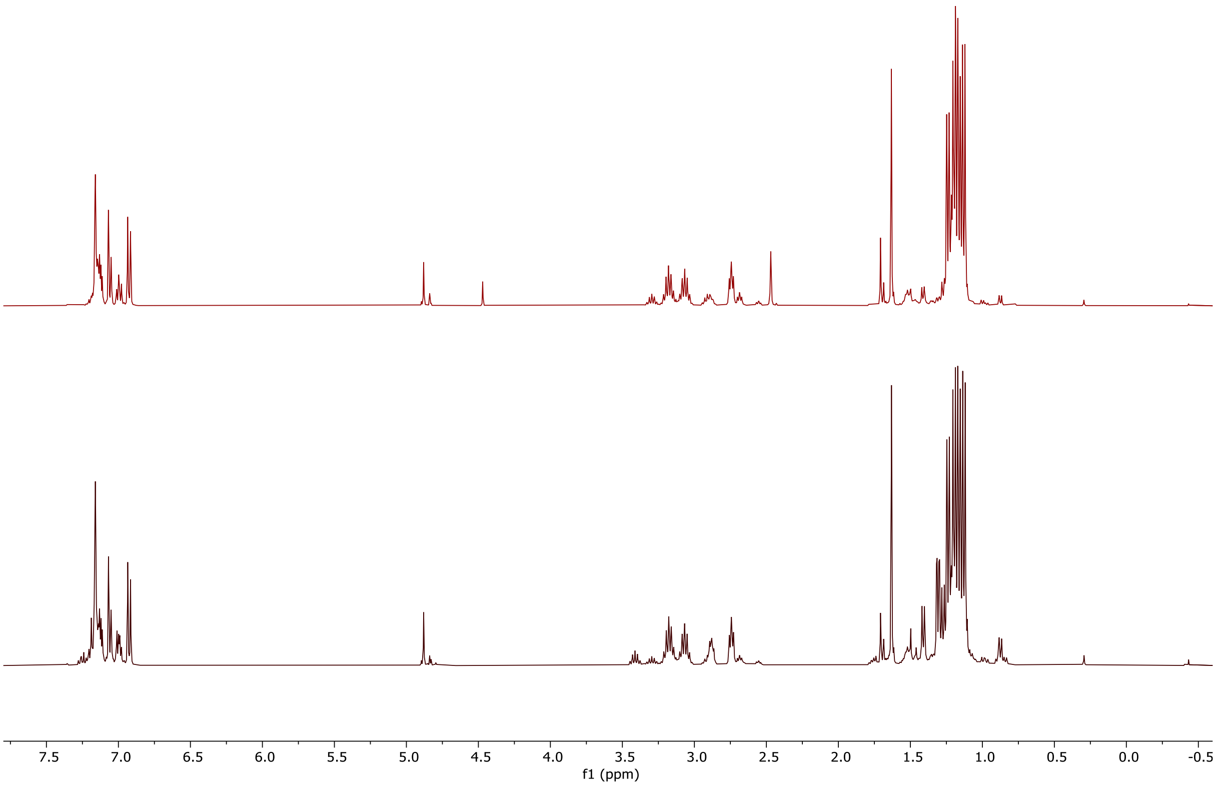

Supplementary Fig. 18 **Stacked ^{1}H NMR spectrum of [(6-Dipp)Cu{μ-H}$_2$MgL] (3) and [(6-Dipp)Cu{μ-D}$_2$MgL] (3*)**. Top spectrum is of [(6-Dipp)Cu{μ-H}$_2$MgL] (3) and bottom spectrum is of [(6-Dipp)Cu{μ-D}$_2$MgL] (3*).

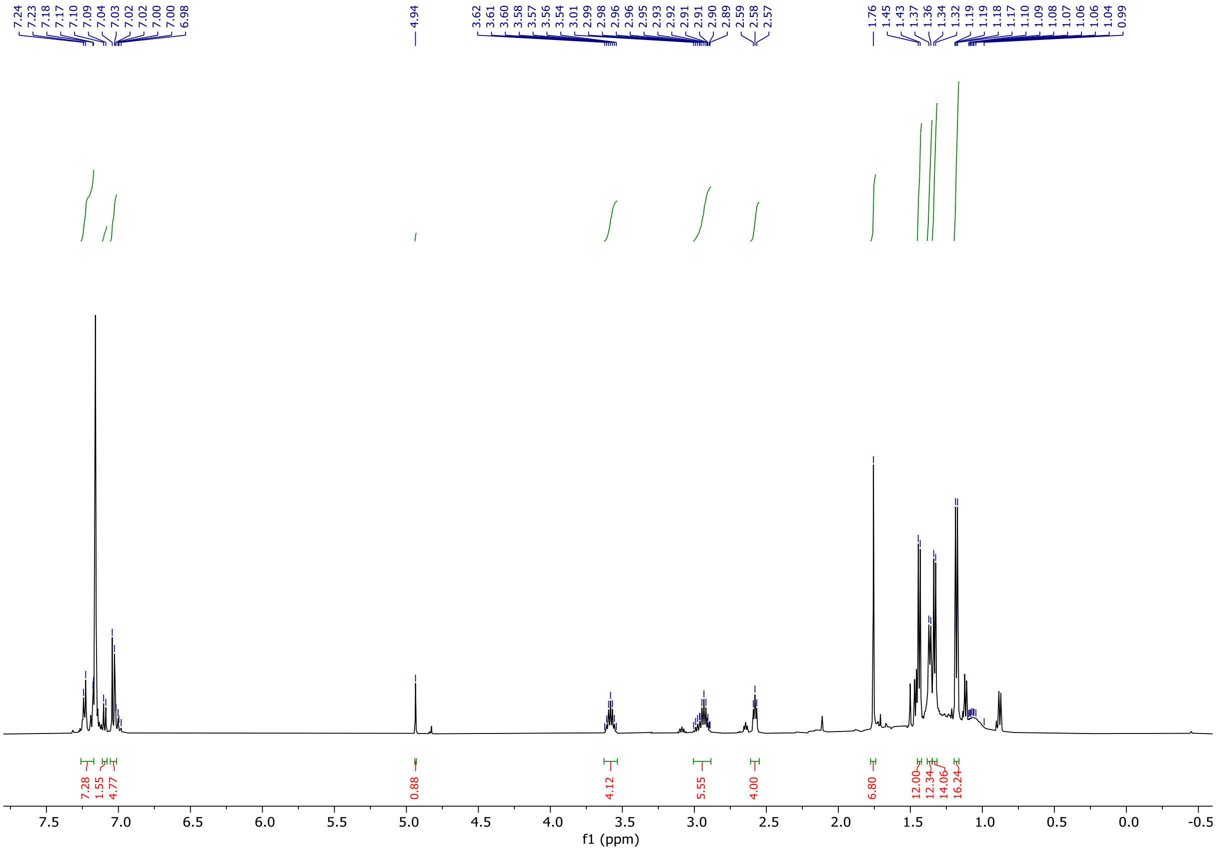

Supplementary Fig. 19 **The ^{1}H NMR spectrum (500 MHz, C_6D_6) of [(6-Dipp)CuC(NCy)$_2$MgL] (4).**

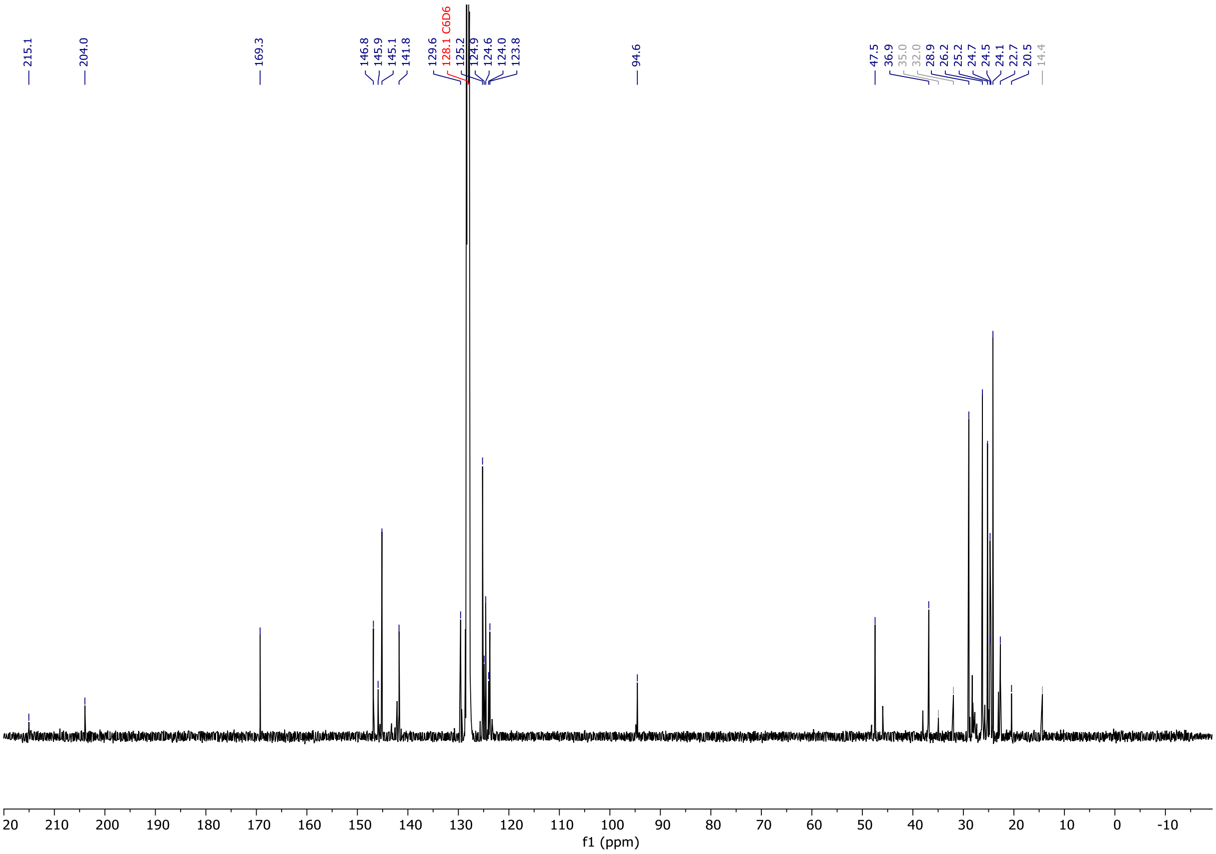

Supplementary Fig. 20 **The $^{13}C\{^{1}H\}$ NMR spectrum (125 MHz, C_6D_6) of [(6-Dipp)CuC(NCy)$_2$MgL] (4).**

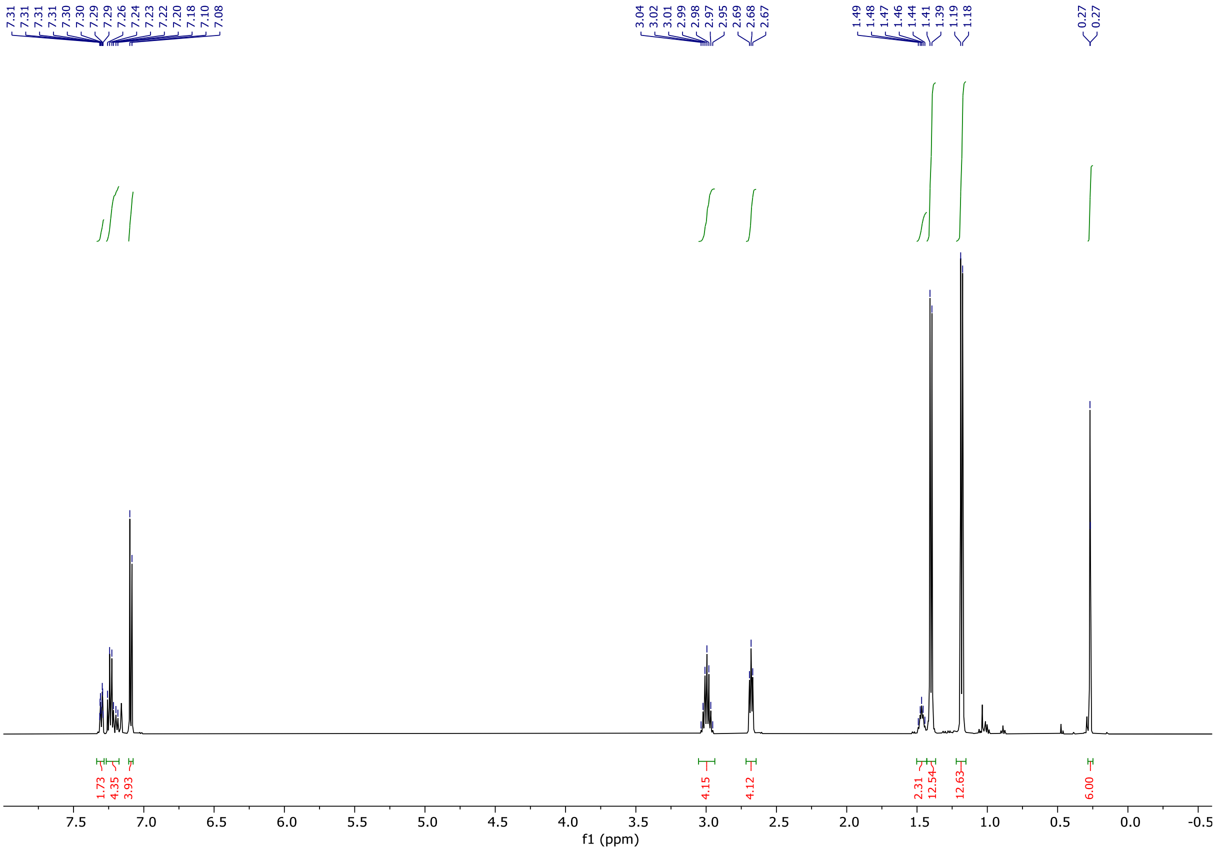

Supplementary Fig. 21 **The ^{1}H NMR spectrum (500 MHz, C_6D_6) of [(6-Dipp)CuSiMe$_2$Ph] (5).**

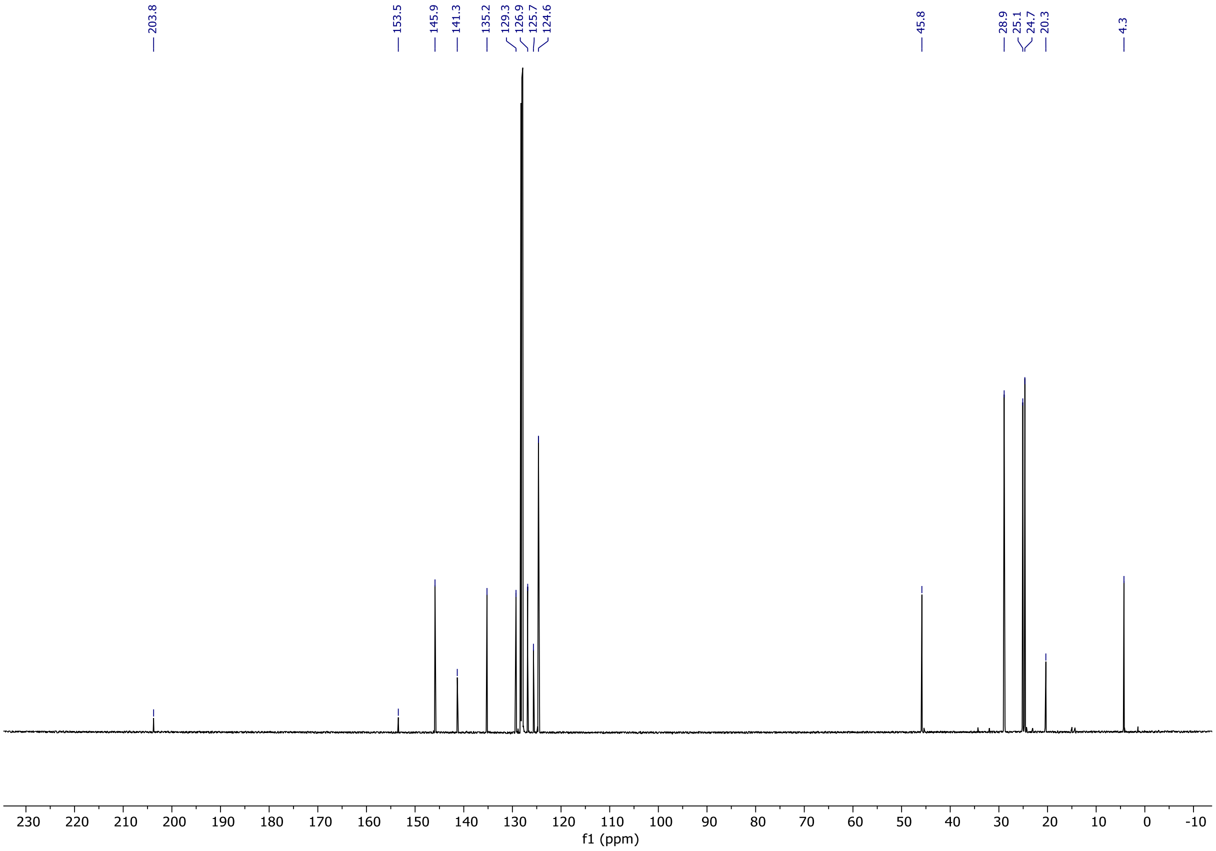

Supplementary Fig. 22 **The $^{13}C\{^{1}H\}$ NMR spectrum (125 MHz, C_6D_6) of [(6-Dipp)CuSiMe$_2$Ph] (5).**

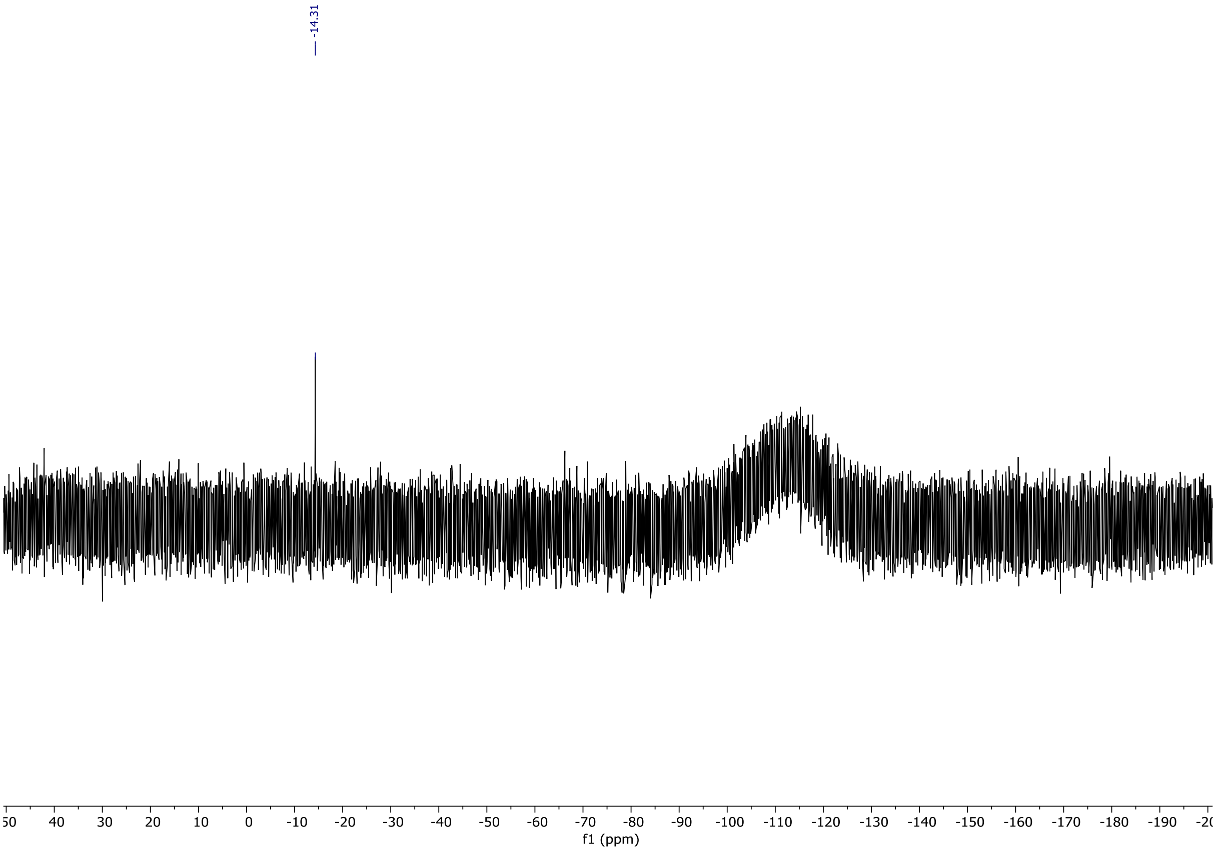

Supplementary Fig. 23 **The ^{29}Si NMR spectrum (99 MHz, C_6D_6) of [(6-Dipp)CuSiMe$_2$Ph] (5).**

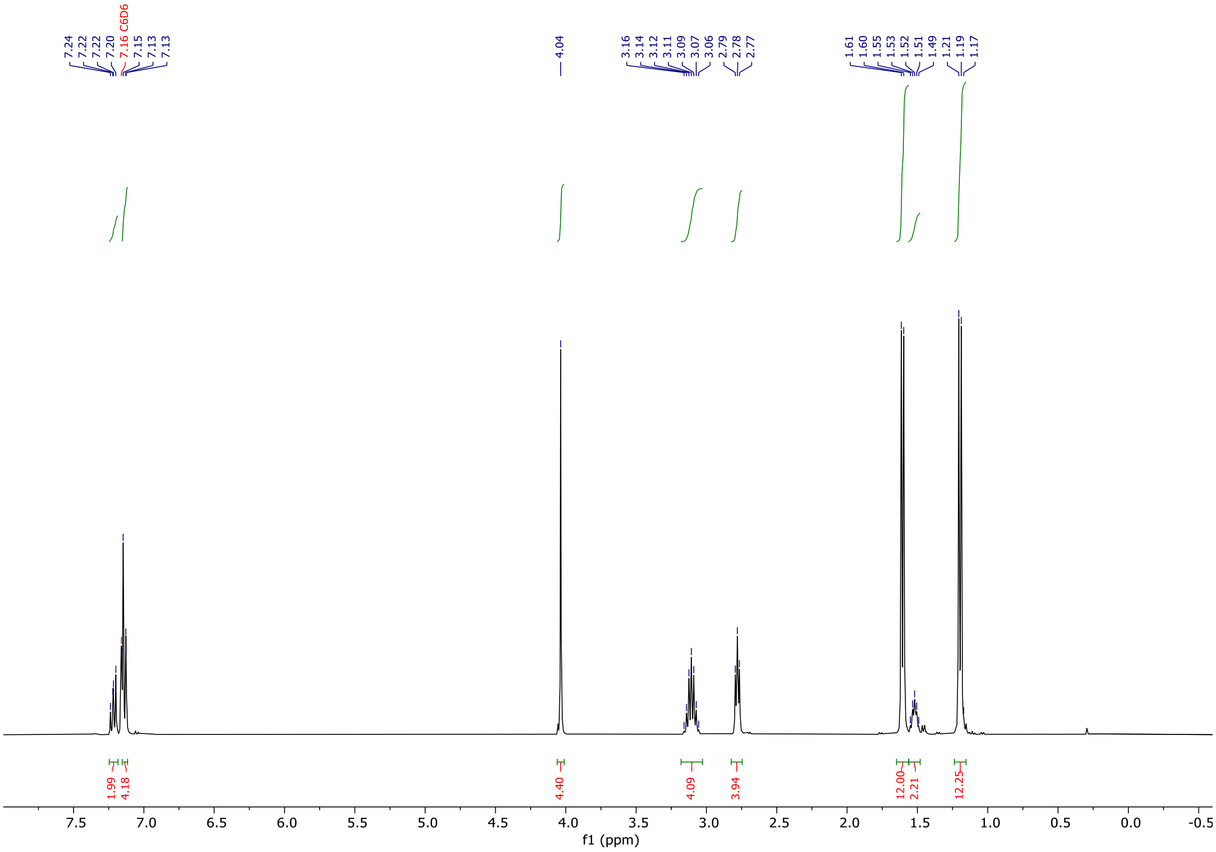

Supplementary Fig. 24 **The ^{1}H NMR spectrum (400 MHz, C_6D_6) of [(6-Dipp)CuFeCp(CO)$_2$] (6).**

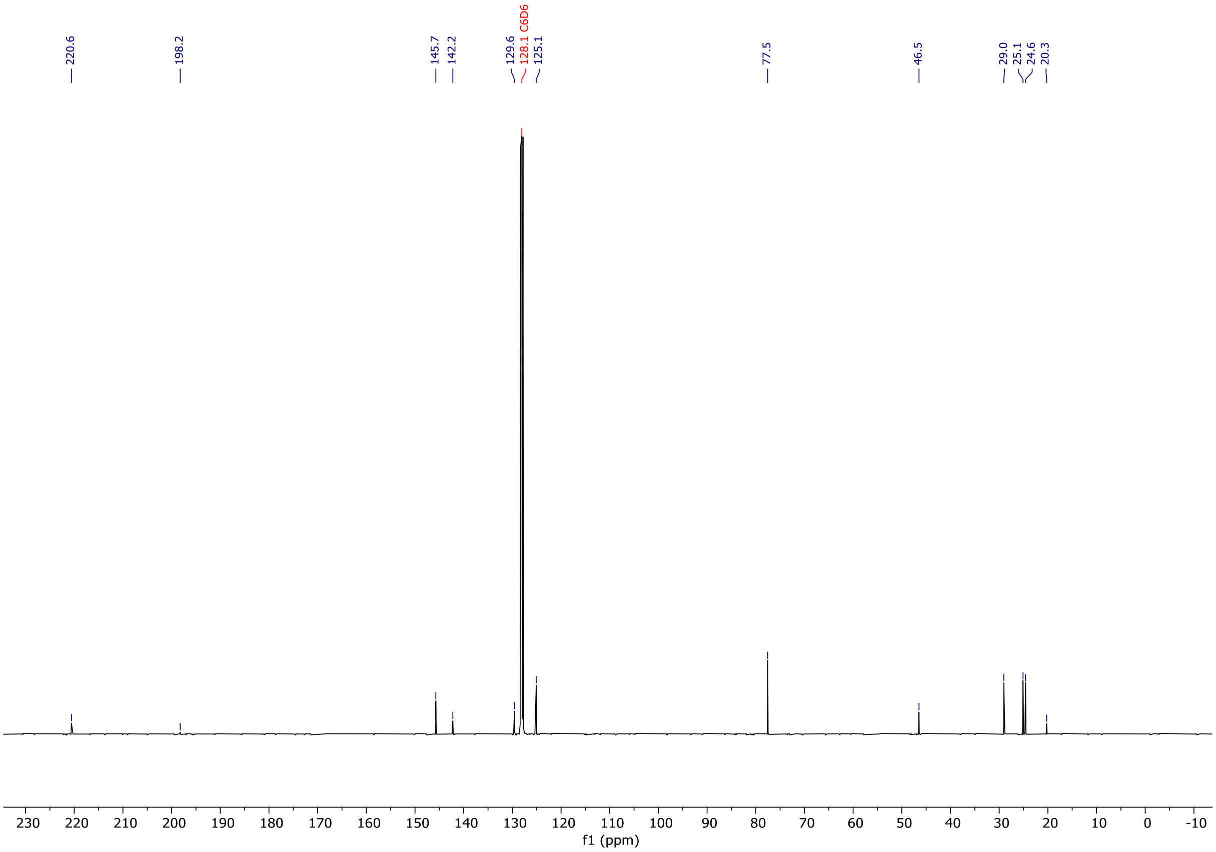

Supplementary Fig. 25 **The $^{13}C\{^{1}H\}$ NMR spectrum (100 MHz, C_6D_6) of [(6-Dipp)CuFeCp(CO)$_2$] (6).**

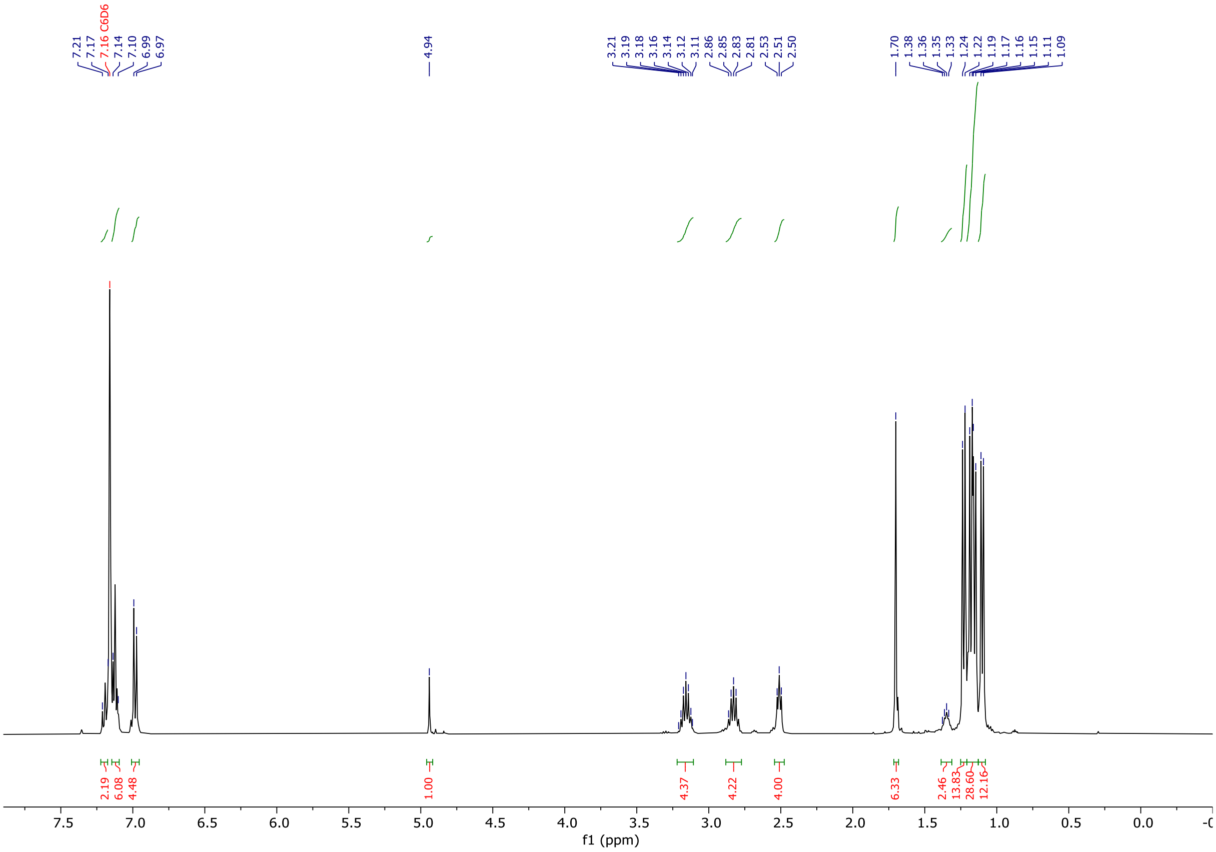

Supplementary Fig. 26 **The ^{1}H NMR spectrum (400 MHz, C_6D_6) of [(6-Dipp)CuZnL] (7).**

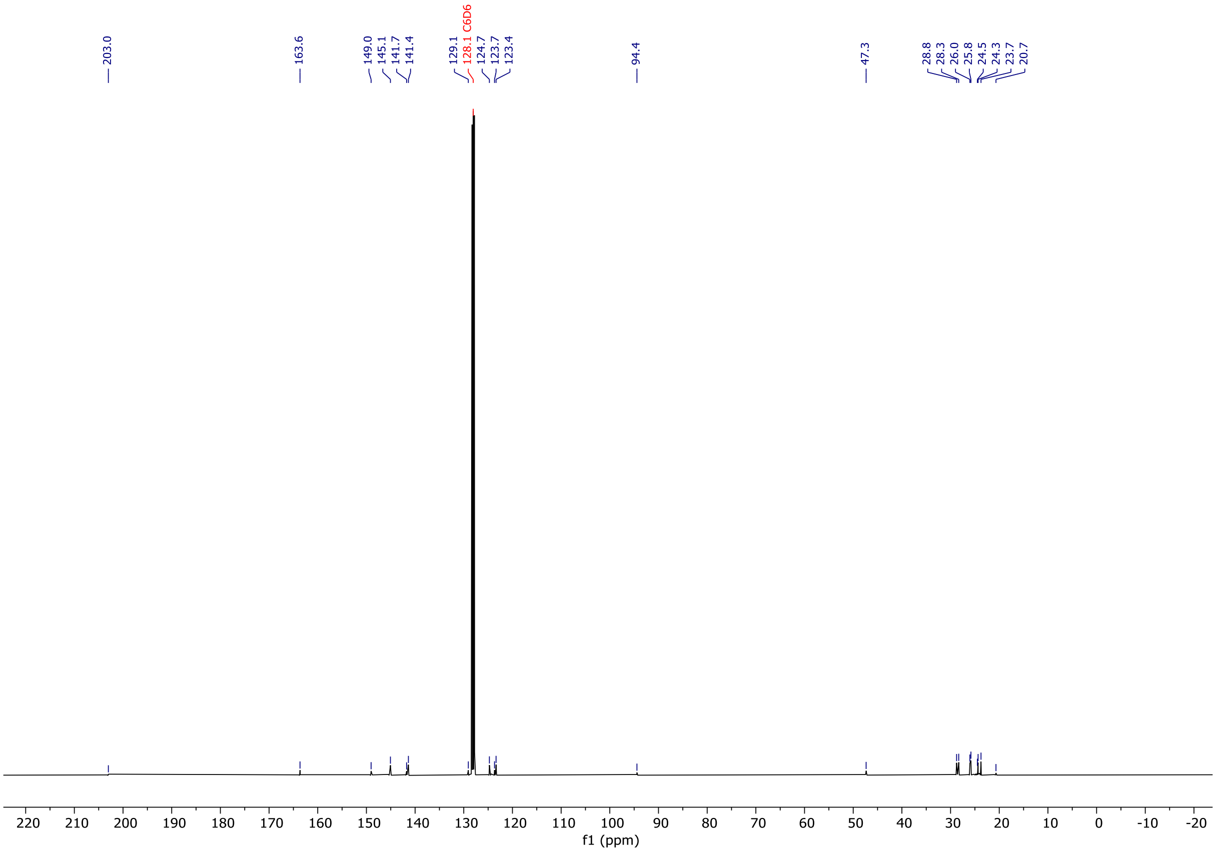

Supplementary Fig. 27 **The $^{13}C\{^{1}H\}$ NMR spectrum (100 MHz, C_6D_6) of [(6-Dipp)CuZnL] (7).**

NMR Scale Reactions

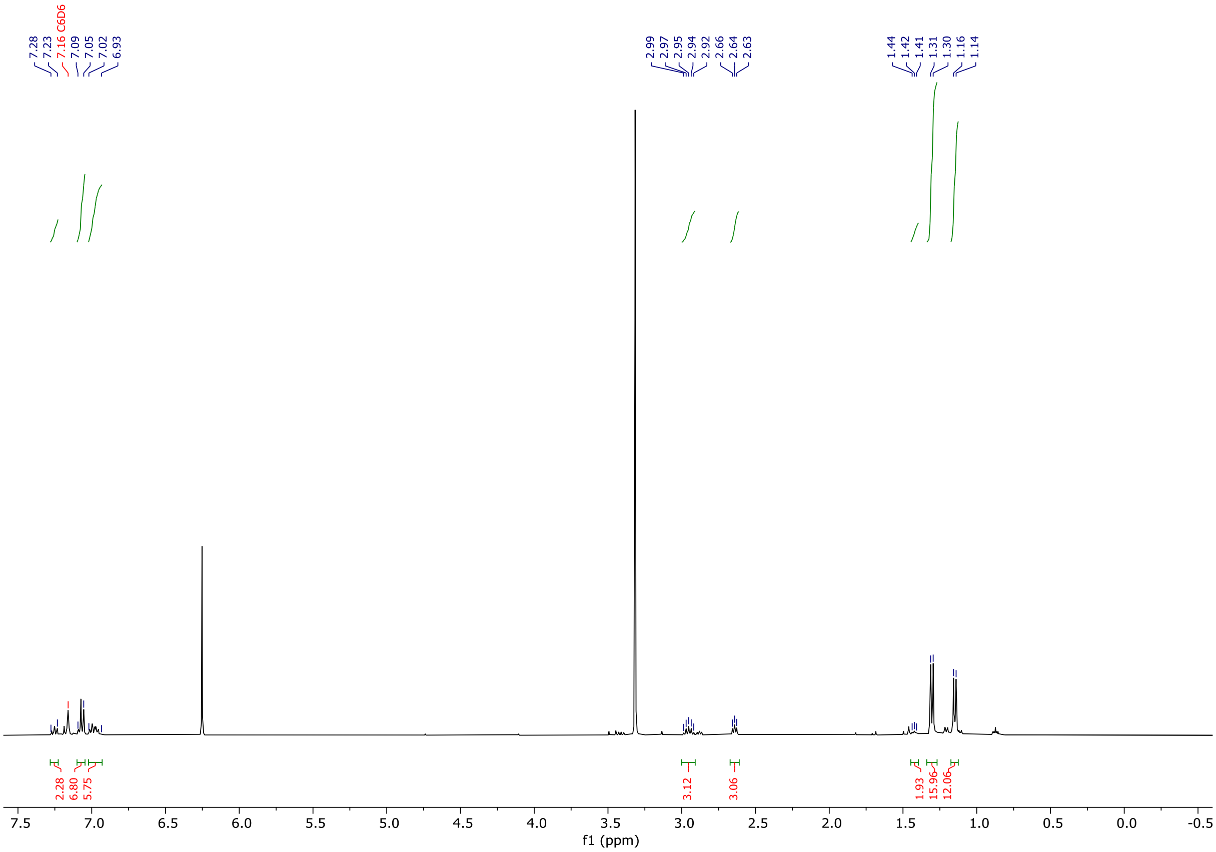

Supplementary Fig. 28 **The *in-situ* ^{1}H NMR spectrum (400 MHz, C_6D_6) of 2 with Ph_2POMe.** Calibrant 1,3,5-trimethoxybenzene present.

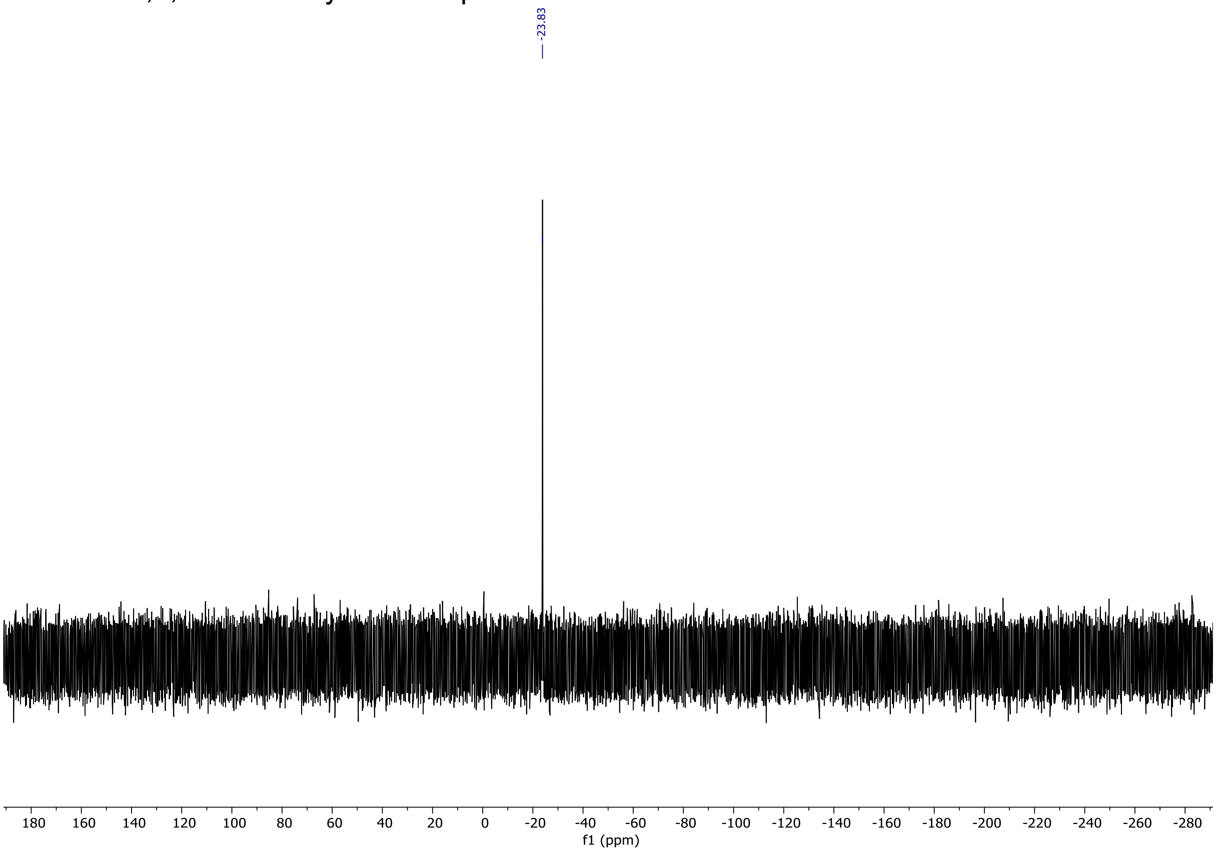

Supplementary Fig. 29 **The *in-situ* ^{31}P NMR spectrum (162 MHz, C_6D_6) of 2 with Ph_2POMe.**

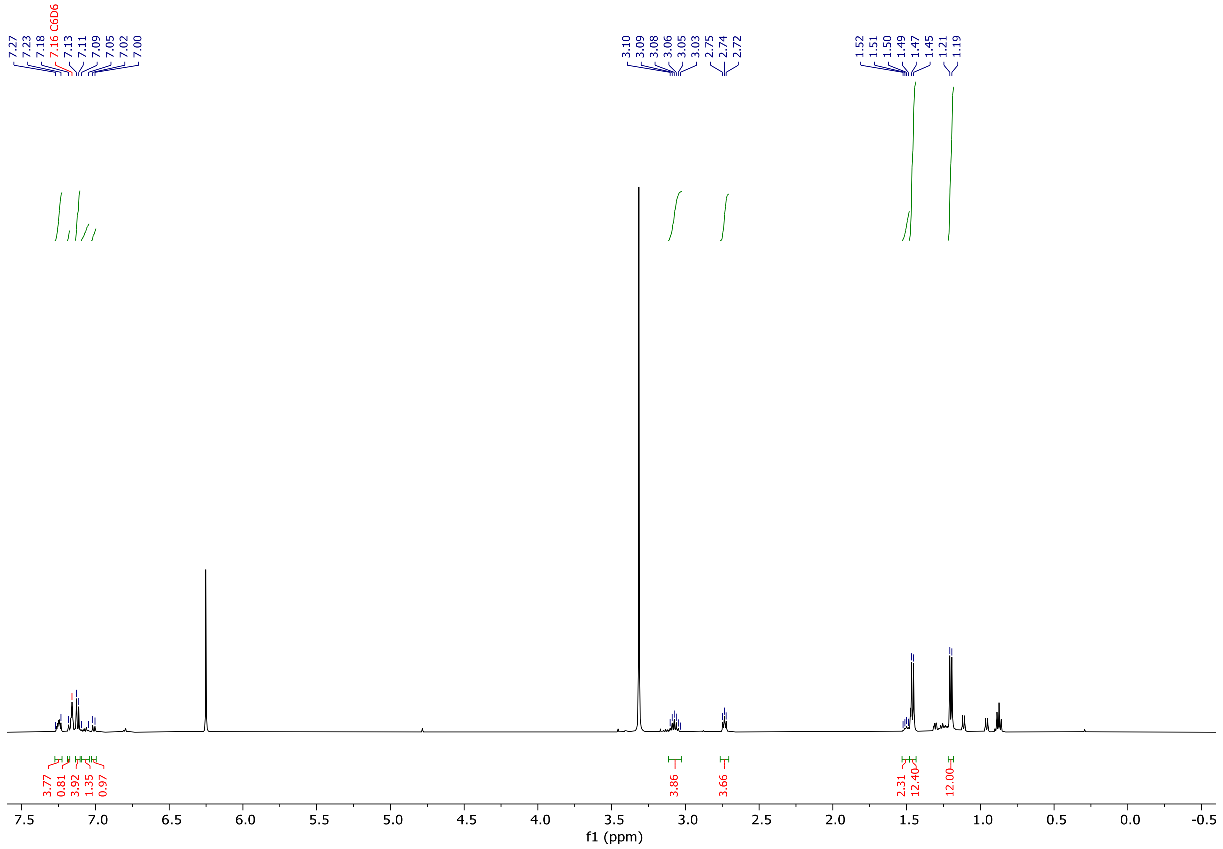

Supplementary Fig. 30 **The *in-situ* ^{1}H NMR spectrum (400 MHz, C_6D_6) of 2 with PhCl.** Calibrant 1,3,5-trimethoxybenzene present.

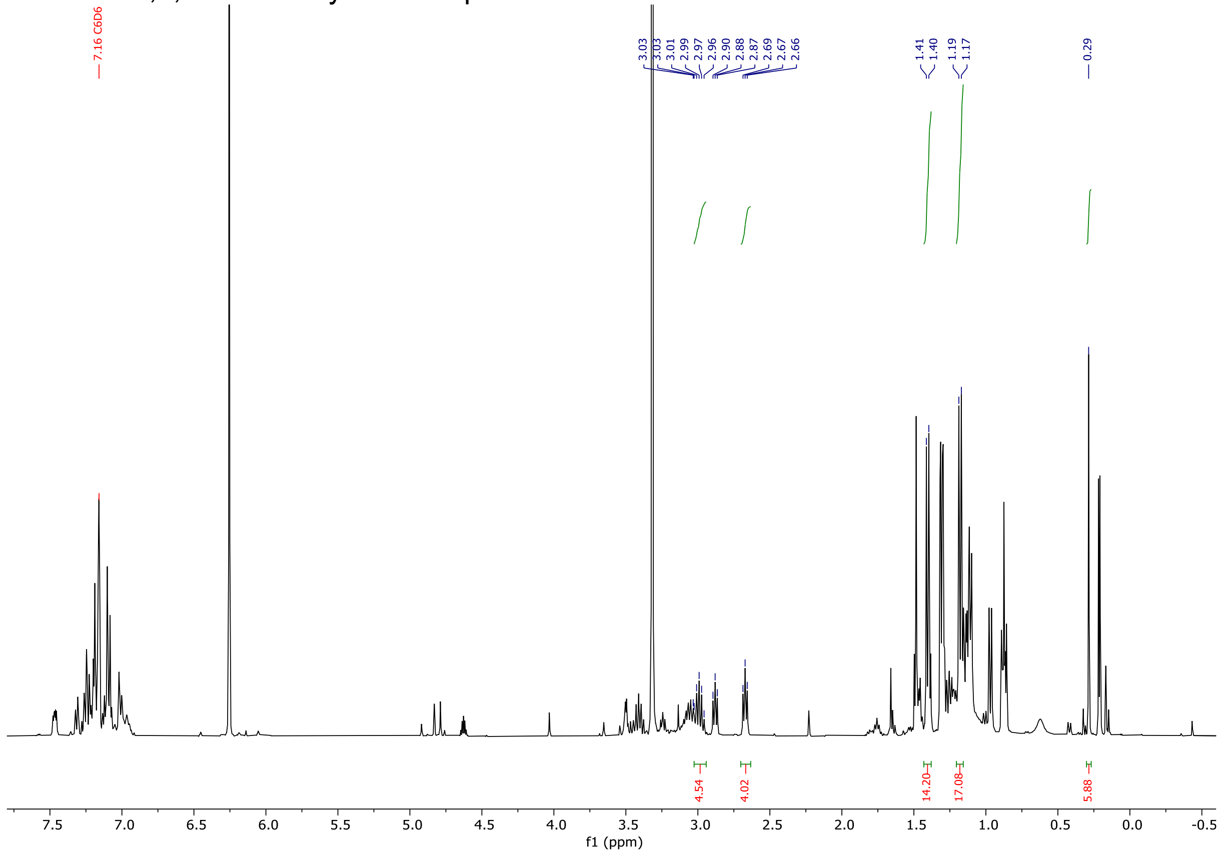

Supplementary Fig. 31 **The *in-situ* ^{1}H NMR spectrum (400 MHz, C_6D_6) of 2 with $HSiMe_2Ph$.** Calibrant 1,3,5-trimethoxybenzene present.

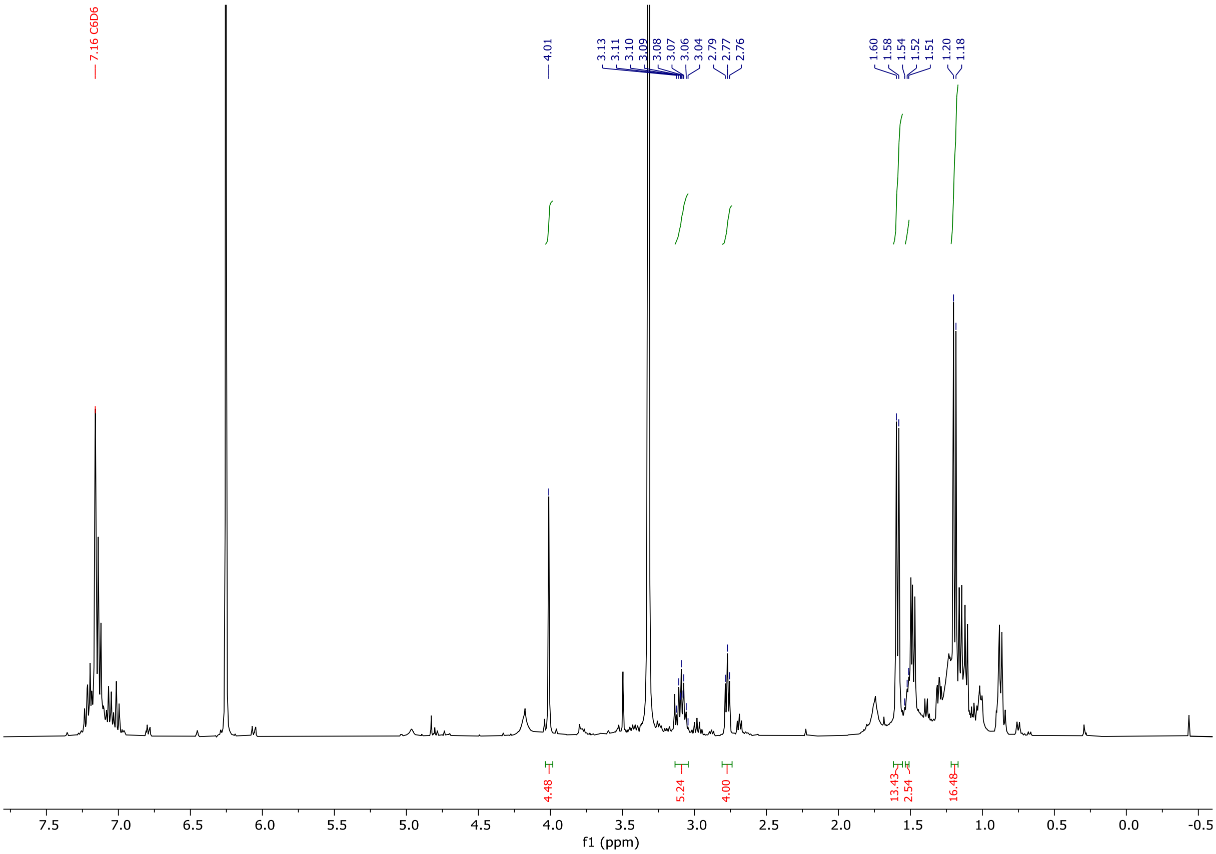

Supplementary Fig. 32 **The *in-situ* ^{1}H NMR spectrum (400 MHz, C_6D_6) of 2 with CpFe(CO)$_2$I.** Calibrant 1,3,5-trimethoxybenzene present.

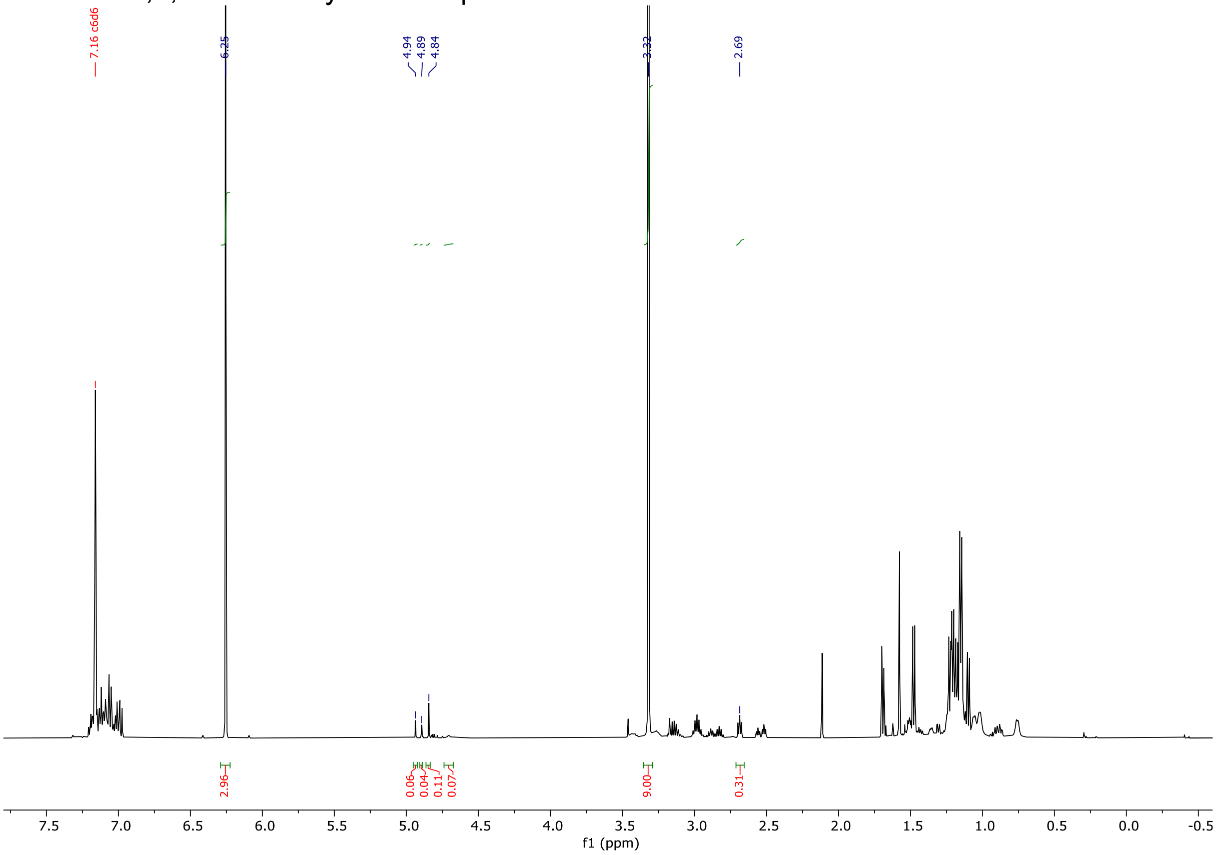

Supplementary Fig. 33 **The *in-situ* calibrated ^{1}H NMR spectrum (500 MHz, C_6D_6) of 2 with 1 eq of LZnI.** Resonances used in the calculations of masses are picked only.

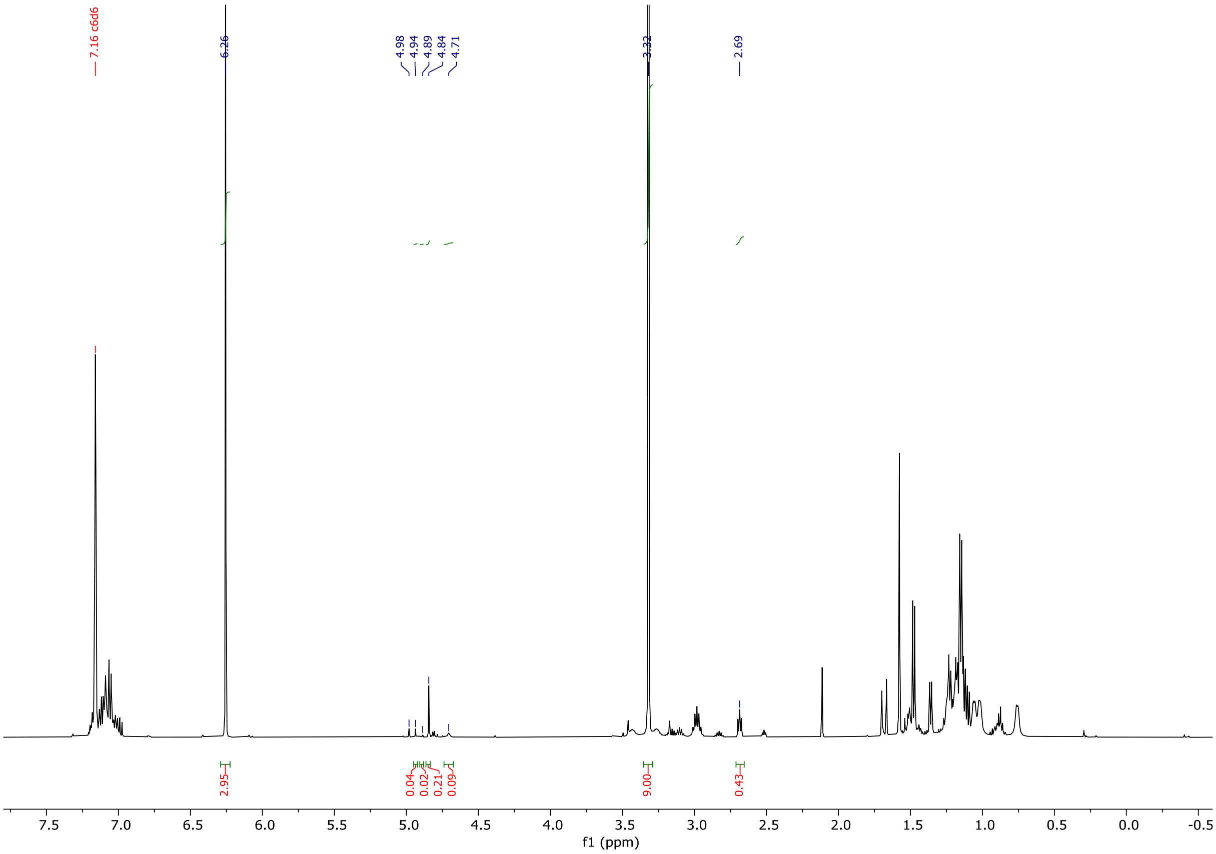

Supplementary Fig. 34 **The *in-situ* calibrated ^{1}H NMR spectrum (500 MHz, C_6D_6) of 2 with 2 eq of LZnI.** Resonances used in the calculations of masses are picked only.

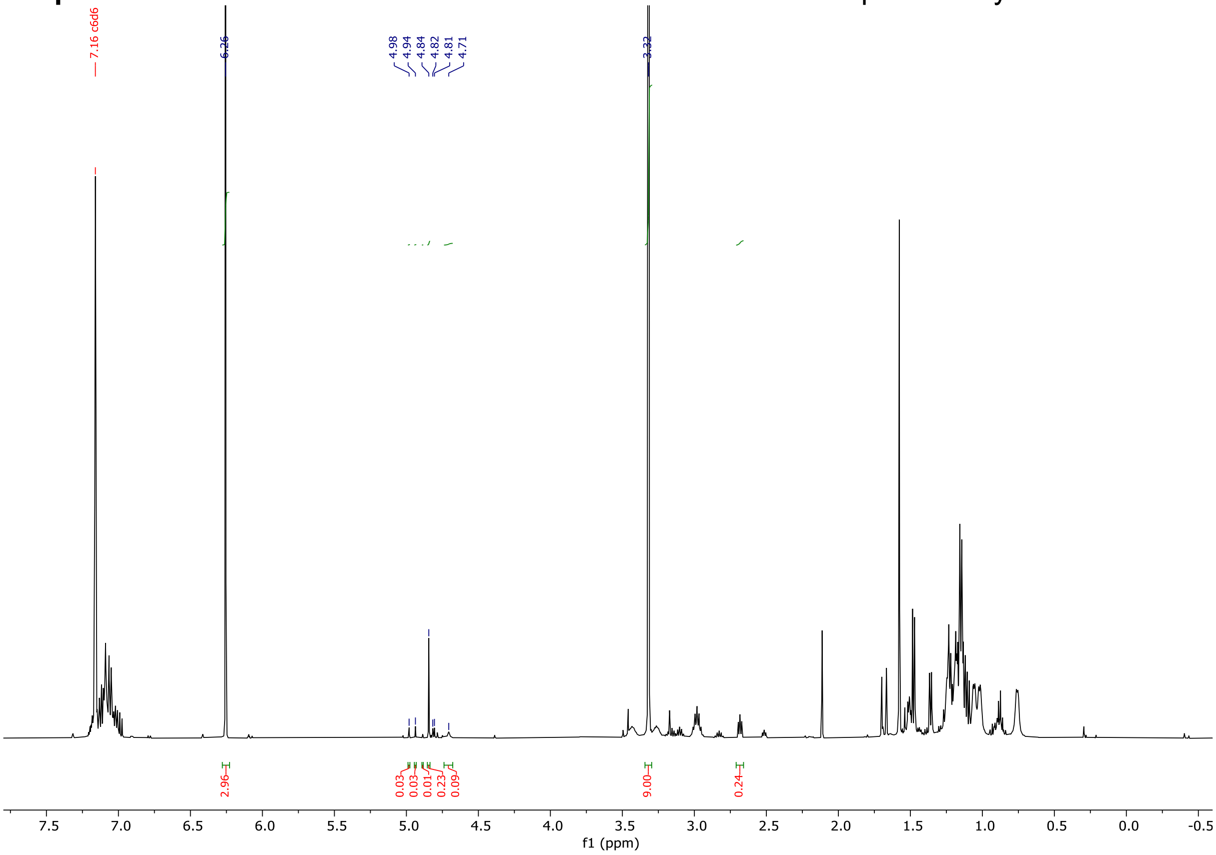

Supplementary Fig. 35 **The *in-situ* calibrated ^{1}H NMR spectrum (500 MHz, C_6D_6) of 2 with 2 eq of LZnI after 62 hours at RT.** Resonances used in the calculations of masses are picked only.

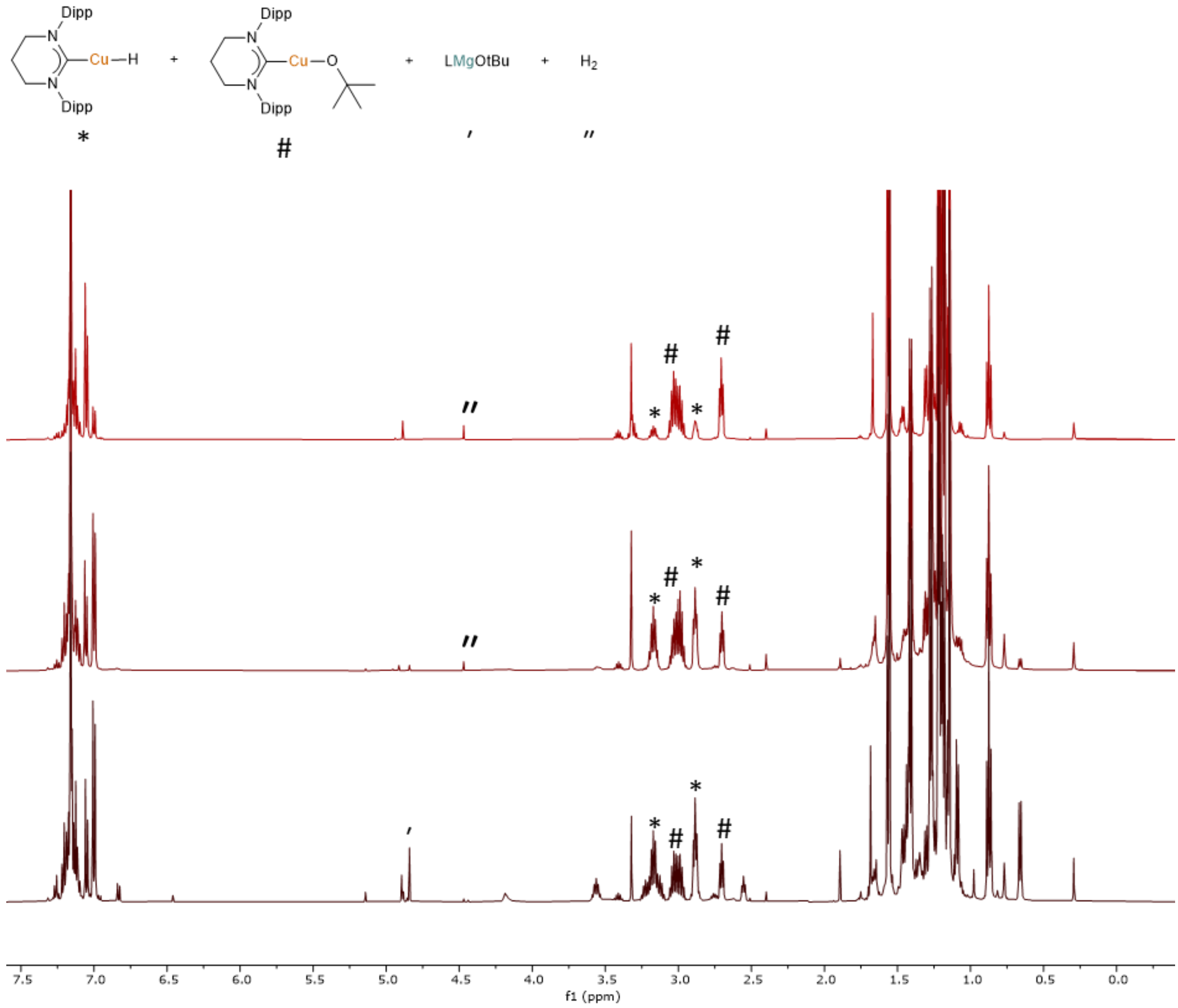

Supplementary Fig. 36 **The *in-situ* calibrated ^{1}H NMR spectrum (500 MHz, C_6D_6) of 2 with ^{t}BuOH.** Bottom spectrum: 1 eq of ^{t}BuOH. Middle spectrum: additional 2 eq of ^{t}BuOH. Top spectrum: After 48 hours at RT. * = (6-Dipp)CuH. # = (6-Dipp)CuO^{t}Bu. ′ = LMgO^{t}Bu. ″ = H_2.

IR Spectra

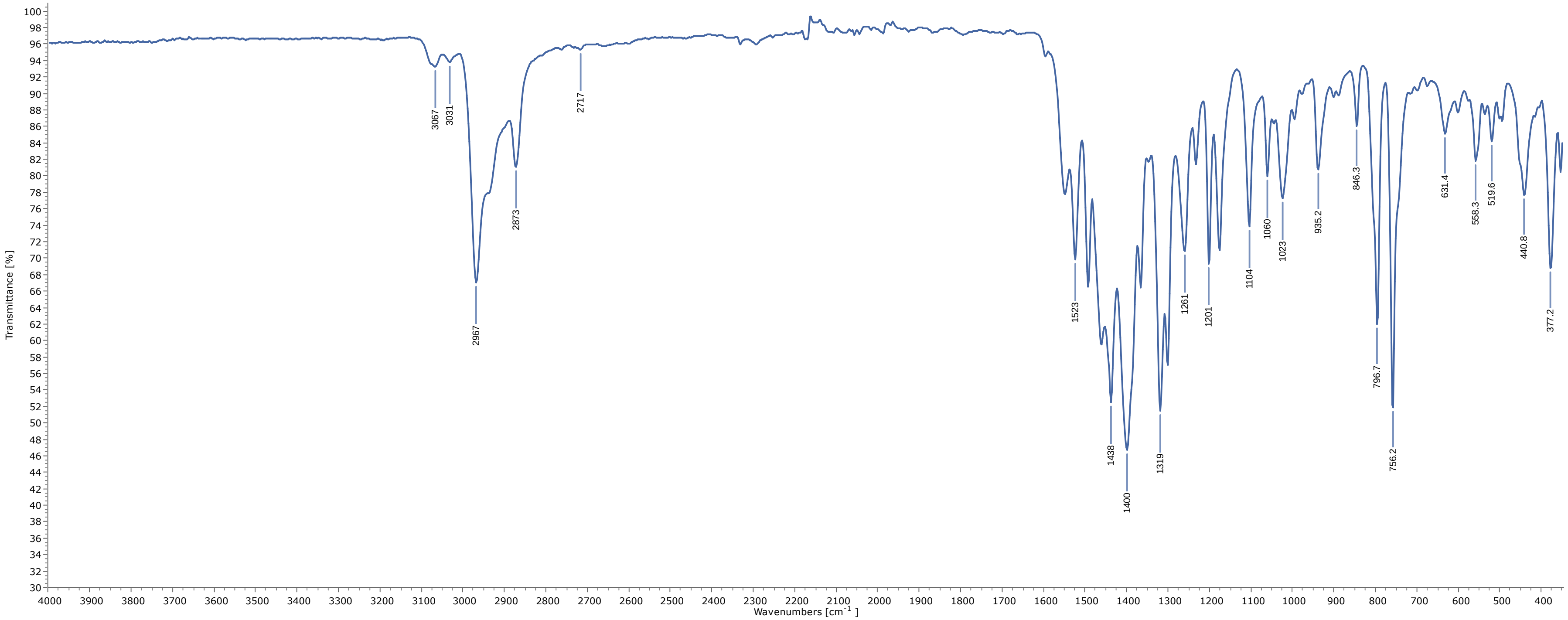

Supplementary Fig. 37 **The IR spectrum of [(6-Dipp)CuMgL] (2).**

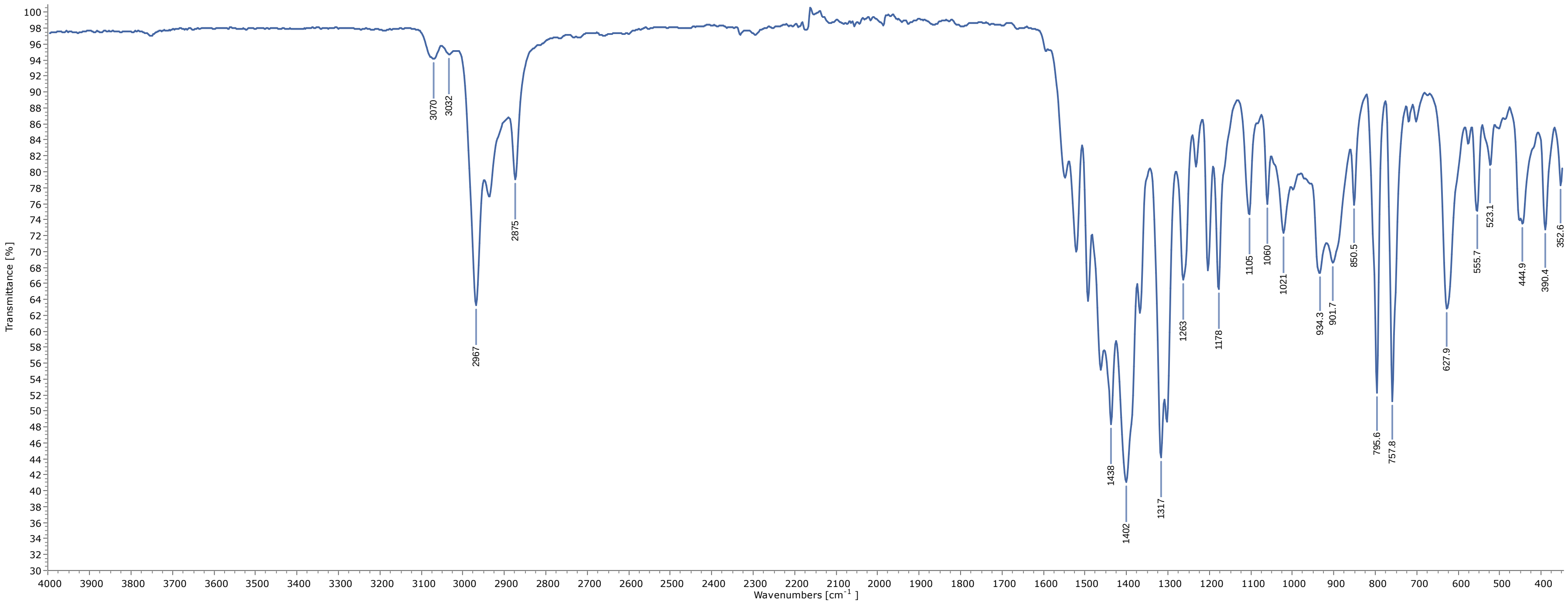

Supplementary Fig. 38 **The IR spectrum of [(6-Dipp)Cu{μ-H}$_2$MgL] (3).**

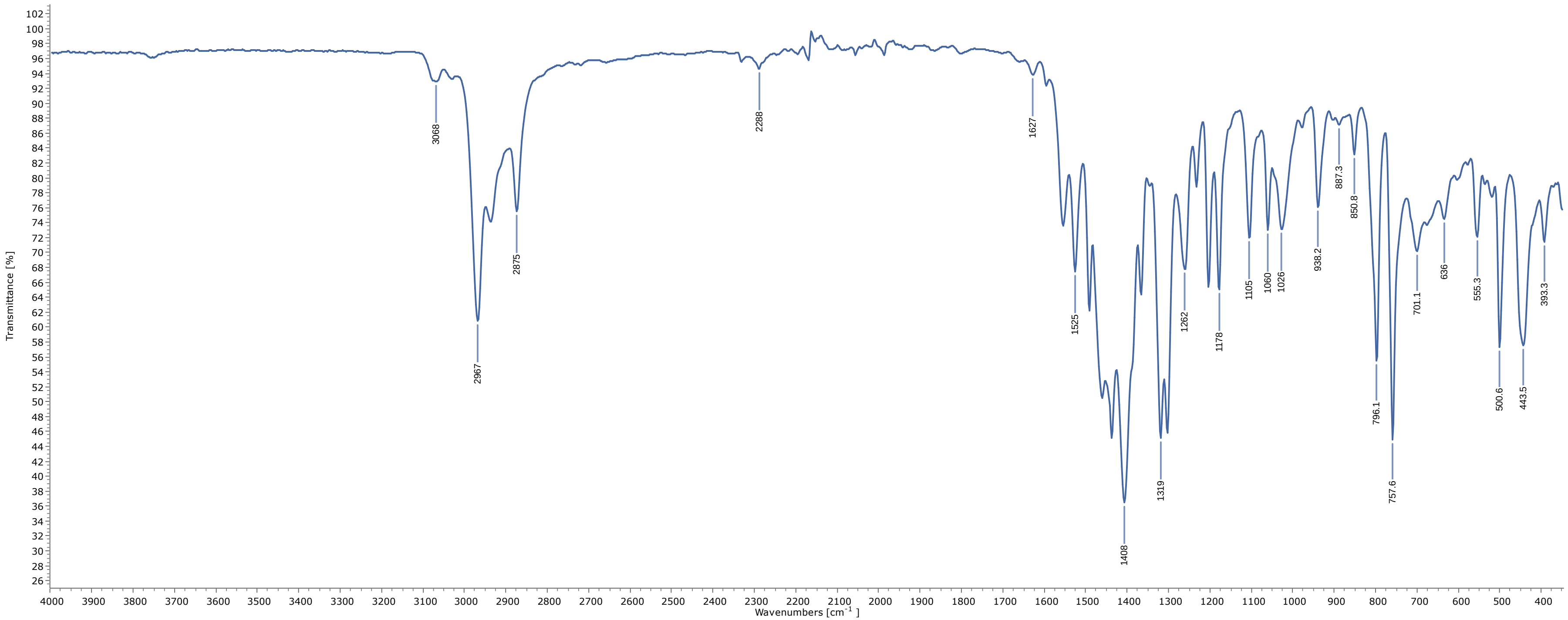

Supplementary Fig. 39 **The IR spectrum of 3* [(6-Dipp)Cu{μ-D}$_2$MgL].** Synthesised identically to [(6-Dipp)Cu{μ-H}$_2$MgL] (3) using D_2 gas in lieu of H_2 gas.

Supplementary Note 4

Computational Details

DFT calculations were performed with Gaussian 16 (C.01).[10] Initial optimizations were performed at the TPSS-D3BJ[11] /def2-SVP[12] level of theory using the 'grid = ultrafine' option, with all stationary points being fully characterized via analytical frequency calculations as minima or transition states (all positive eigenvalues or one imaginary eigenvalue respectively). Note that species **3** yields a small additional imaginary frequency of -3.1 cm^{-1} , which remained after various efforts to remove it were undertaken. To yield the reaction free-energy profiles for hydrogen addition and activation, energies were recomputed at the PBE0-D3BJ[13] (CPCM=C_6H_6)/def2-TZVPP level using ORCA version 5.0.2[14] with the CPCM model employed to incorporate benzene solvation effects (ε = 2.2706 The Quantum Theory of Atoms in Molecules[15] and Natural Bonding Orbital[16] analyses were performed on the TPSS-D3BJ optimized geometries at the PBE0-D3BJ/def2-TZVPP level of theory. Contour plots were generated in the AIMStudio package using critical point (CP) visualisation threshold values of 0.025.

To generate the 10 additional structures alongside **2**, **TS** and **3** for further charge analysis with QTAIM, the Intrinsic Reaction Coordinate procedure was employed in Gaussian 16, with five structures taken in the reverse (labelled **R1** – **R5**) and forward (labelled **F1** – **F5**) directions each, where the local quadratic approximation was employed in the predictor step.

QTAIM Contour Plots & Tabulated Data

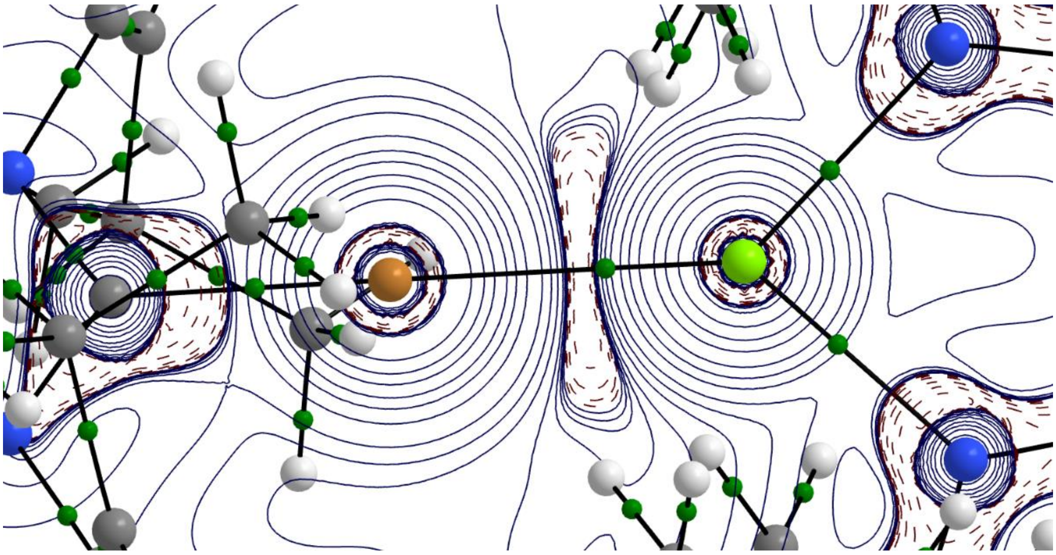

Supplementary Fig. 40 **Contour plot of the Laplacian, ($\nabla^2\rho(r)$), of 2.**

Supplementary Table 6 **Selected BCP data for 2.**

Atoms	ρ(r)	$\nabla^2\rho(r)$	Ellipticity	V	G	H	DI(A|B)
Cu1 - Mg2	0.037113	0.039101	0.080384	-0.02781	0.01879	-0.00902	0.473425

Supplementary Table 7 **Selected QTAIM and NBO atomic data for 2**

Atom	L(A)	N(A)	% Loc(A)	q(r) (AIM)	q(r) (NBO)
Cu1	-0.00037	29.517224	96.09455	-0.517224	-0.08022
Mg2	-7.6E-05	10.548824	95.07643	1.451176	1.09993

MO and NBO Data

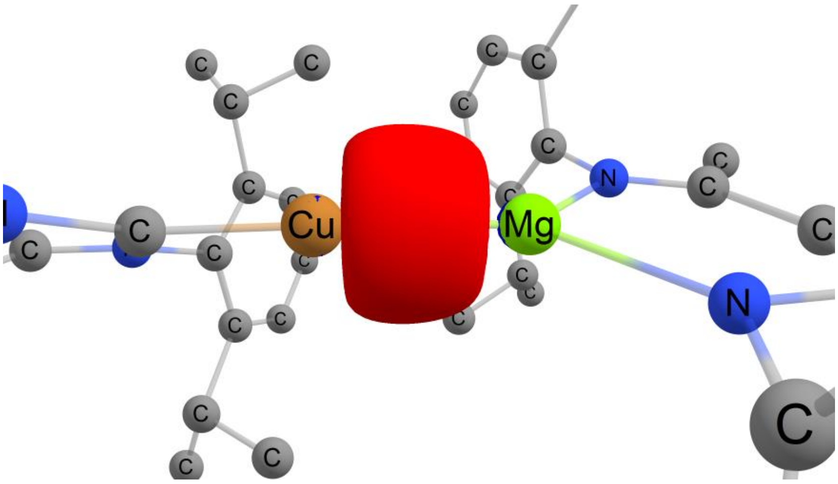

Supplementary Fig. 41 **Natural Localized Molecular Orbital of the Cu–Mg bond in 2.**

Supplementary Table 8 **Atomic contributions to the $\sigma_{Cu\text{–}B}$ bond NLMO in 2**.

Atom	Contribution to NLMO in % (contributing s, p and d character)
Cu	53.5 (95.1 % s character, 3.7 % d)
Mg	39.0 (97.5 % s character, 2.4 % p)

(a) **HOMO** (-4.0 eV) (b) **LUMO** (-0.6 eV)

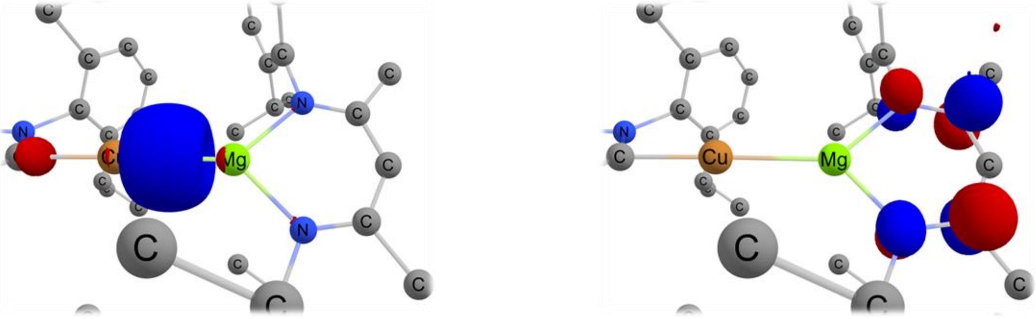

Supplementary Fig. 42 **Frontier molecular orbitals of 2**. **a** The HOMO of compound **2**. **b** The LUMO of compound **2**. Reported energies (in eV).

IRC-Charge analysis

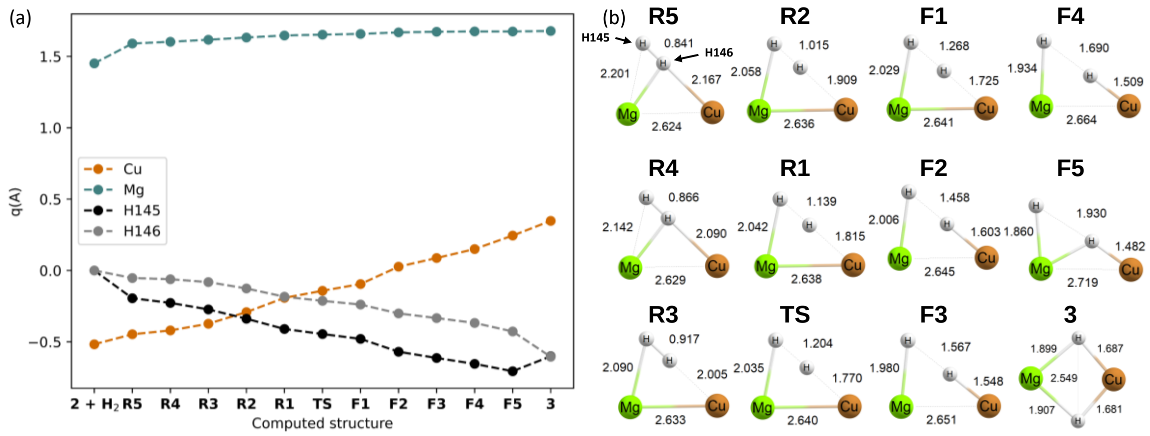

Supplementary Fig. 43 **IRC-Charge analysis of the reaction of compound 2 with H_2. a** Evolution of computed atomic charges (with AIM) across the reaction profile at the Cu (orange), Mg (green) and H (black/grey) centres (PBE0-D3BJ(CPCM=C6H6)/def2-TZVPP//TPSS-D3BJ/def2-SVP level of theory). **2**. **b** Structures and selected interatomic distances of the {$CuMgH_2$} moiety in all structures.

Supplementary Table 9 **Computed atomic charges, q(A) with QTAIM and NBO for structures in the IRC-Charge analysis.** Tabulated data determined from the IRC-charge analysis illustrated in Figure S43.

Structure	Name (#)	q(A)	
		AIM	NBO
**2**	Cu(1)	-0.51722	-0.08022
**2**	Mg(2)	1.451176	1.09993
**2**	H(145)	0	0
**2**	H(146)	0	0
**R5**	Cu(1)	-0.44693	-0.08779
**R5**	Mg(2)	1.590398	1.43091
**R5**	H(145)	-0.19445	-0.21533
**R5**	H(146)	-0.0528	-0.12048
**R4**	Cu(1)	-0.41995	-0.07364
**R4**	Mg(2)	1.602594	1.46948
**R4**	H(145)	-0.2271	-0.25232
**R4**	H(146)	-0.06131	-0.1403
**R3**	Cu(1)	-0.37318	-0.04315
**R3**	Mg(2)	1.616793	1.51608
**R3**	H(145)	-0.27283	-0.30492
**R3**	H(146)	-0.08143	-0.17478
**R2**	Cu(1)	-0.29176	0.01676
**R2**	Mg(2)	1.632321	1.57075
**R2**	H(145)	-0.33821	-0.38289
**R2**	H(146)	-0.12618	-0.23205
**R1**	Cu(1)	-0.19184	0.09357
**R1**	Mg(2)	1.646405	1.6187
**R1**	H(145)	-0.41002	-0.4714
**R1**	H(146)	-0.18403	-0.29442
**TS**	Cu(1)	-0.14234	0.13275
**TS**	Mg(2)	1.652395	1.63785
**TS**	H(145)	-0.44521	-0.51487
**TS**	H(146)	-0.21279	-0.32242

Structure	Name (#)	q(A)	
		AIM	NBO
**TS**	Cu(1)	-0.14234	0.13275
**TS**	Mg(2)	1.652395	1.63785
**TS**	H(145)	-0.44521	-0.51487
**TS**	H(146)	-0.21279	-0.32242
**F1**	Cu(1)	-0.09508	0.17097
**F1**	Mg(2)	1.657533	1.65394
**F1**	H(145)	-0.47941	-0.5568
**F1**	H(146)	-0.23866	-0.34761
**F2**	Cu(1)	0.026372	0.27188
**F2**	Mg(2)	1.668197	1.68361
**F2**	H(145)	-0.56942	-0.66436
**F2**	H(146)	-0.30103	-0.40545
**F3**	Cu(1)	0.087551	0.32143
**F3**	Mg(2)	1.672809	1.69021
**F3**	H(145)	-0.61302	-0.71367
**F3**	H(146)	-0.33364	-0.43092
**F4**	Cu(1)	0.149959	0.36976
**F4**	Mg(2)	1.674755	1.68992
**F4**	H(145)	-0.65447	-0.75645
**F4**	H(146)	-0.3675	-0.45538
**F5**	Cu(1)	0.244617	0.44192
**F5**	Mg(2)	1.674705	1.67721
**F5**	H(145)	-0.70615	-0.7991
**F5**	H(146)	-0.4272	-0.49576
**3**	Cu(1)	0.347725	0.64389
**3**	Mg(2)	1.678113	1.70009
**3**	H(145)	-0.60113	-0.71875
**3**	H(146)	-0.60469	-0.72422

References

1. Hicks, J., Juckel, M., Paparo, A., Dange, D., Jones, C. Multigram Syntheses of Magnesium(I) Compounds Using Alkali Metal Halide Supported Alkali Metals as Dispersible Reducing Agents. *Organometallics* **37**, 4810-4813 (2018).

2. Blake, M. P., Kaltsoyannis, N., Mountford, P. Synthesis, molecular and electronic structure, and reactions of a Zn–Hg–Zn bonded complex. *Chem. Commun.* **51**, 5743-5746 (2015).

3. Babula, D. J., Charman, R. S. C., Hobson, J. A., Mahon, M. F., Liptrot, D. J. Dial-a-base mechanochemical synthesis of N-heterocyclic carbene copper complexes. *Dalton Trans.* **53**, 3990-3993 (2024).

4. Plotzitzka, J., Kleeberg, C. [(NHC)CuI–ER3] Complexes (ER3 = SiMe2Ph, SiPh3, SnMe3): From Linear, Mononuclear Complexes to Polynuclear Complexes with Ultrashort CuI···CuI Distances. *Inorg. Chem.* **55**, 4813-4823 (2016).

5. Jayarathne, U., Parmelee, S. R., Mankad, N. P. Small Molecule Activation Chemistry of Cu–Fe Heterobimetallic Complexes Toward CS2 and N2O. *Inorg. Chem.* **53**, 7730-7737 (2014).

6. Horsley Downie, T. M.*, et al.* The first ring-expanded NHC–copper(i) phosphides as catalysts in the highly selective hydrophosphination of isocyanates. *Chem. Commun.* **56**, 13359-13362 (2020).

7. Sheldrick, G. SHELXT - Integrated space-group and crystal-structure determination. *Acta Cryst. A* **71**, 3-8 (2015).

8. Sheldrick, G. Crystal structure refinement with SHELXL. *Acta Cryst. C* **71**, 3-8 (2015).

9. Dolomanov, O. V., Bourhis, L. J., Gildea, R. J., Howard, J. A. K., Puschmann, H. OLEX2: a complete structure solution, refinement and analysis program. *J. Appl. Crystallogr.* **42**, 339-341 (2009).

10. Frisch, M. J.*, et al.* Gaussian 16 (Rev C.01), Gaussian Inc, Wallingford, CT. (2016).

11. Tao, J., Perdew, J. P., Staroverov, V. N., Scuseria, G. E. Climbing the Density Functional Ladder: Nonempirical Meta--Generalized Gradient Approximation Designed for Molecules and Solids. *Phys. Rev. Lett.* **91**, 146401 (2003).

12. Weigend, F., Ahlrichs, R. Balanced basis sets of split valence, triple zeta valence and quadruple zeta valence quality for H to Rn: Design and assessment of accuracy. *Phys. Chem. Chem. Phys.* **7**, 3297-3305 (2005).

13. Adamo, C., Barone, V. Toward reliable density functional methods without adjustable parameters: The PBE0 model. *J. Chem. Phys.* **110**, 6158-6170 (1999).

14. Neese, F., Wennmohs, F., Becker, U., Riplinger, C. The ORCA quantum chemistry program package. *J. Chem. Phys.* **152**, 224108 (2020).

15. Keith, T. A. AIMAll (Version 19.10. 12). *TK Gristmill Software: Overland Park, KS, USA*23 (2019).

16. Glendening, E. D.*, et al. NBO, Version 7.0. 10; Theoretical Chemistry Institute, University of Wisconsin: Madison, WI, USA, 2018*.
